# Functional connectivity structure of cortical calcium dynamics in anesthetized and awake mice

**DOI:** 10.1371/journal.pone.0185759

**Published:** 2017-10-19

**Authors:** Patrick W. Wright, Lindsey M. Brier, Adam Q. Bauer, Grant A. Baxter, Andrew W. Kraft, Matthew D. Reisman, Annie R. Bice, Abraham Z. Snyder, Jin-Moo Lee, Joseph P. Culver

**Affiliations:** 1 Department of Biomedical Engineering, Washington University in St. Louis, St. Louis, Missouri, United States of America; 2 Department of Radiology, Washington University School of Medicine, St. Louis, Missouri, United States of America; 3 Department of Neuroscience, Washington University in St. Louis, St. Louis, Missouri, United States of America; 4 Department of Physics, Washington University in St. Louis, St. Louis, Missouri, United States of America; 5 Department Neurology, Washington University School of Medicine, St. Louis, Missouri, United States of America; Ghent University, BELGIUM

## Abstract

The interplay between hemodynamic-based markers of cortical activity (e.g. fMRI and optical intrinsic signal imaging), which are an indirect and relatively slow report of neural activity, and underlying synaptic electrical and metabolic activity through neurovascular coupling is a topic of ongoing research and debate. As application of resting state functional connectivity measures is extended further into topics such as brain development, aging and disease, the importance of understanding the fundamental physiological basis for functional connectivity will grow. Here we extend functional connectivity analysis from hemodynamic- to calcium-based imaging. Transgenic mice (n = 7) expressing a fluorescent calcium indicator (GCaMP6) driven by the *Thy1* promoter in glutamatergic neurons were imaged transcranially in both anesthetized (using ketamine/xylazine) and awake states. Sequential LED illumination (λ = 454, 523, 595, 640nm) enabled concurrent imaging of both GCaMP6 fluorescence emission (corrected for hemoglobin absorption) and hemodynamics. Functional connectivity network maps were constructed for infraslow (0.009–0.08Hz), intermediate (0.08–0.4Hz), and high (0.4–4.0Hz) frequency bands. At infraslow and intermediate frequencies, commonly used in BOLD fMRI and fcOIS studies of functional connectivity and implicated in neurovascular coupling mechanisms, GCaMP6 and HbO_2_ functional connectivity structures were in high agreement, both qualitatively and also quantitatively through a measure of spatial similarity. The spontaneous dynamics of both contrasts had the highest correlation when the GCaMP6 signal was delayed with a ~0.6–1.5s temporal offset. Within the higher-frequency delta band, sensitive to slow wave sleep oscillations in non-REM sleep and anesthesia, we evaluate the speed with which the connectivity analysis stabilized and found that the functional connectivity maps captured putative network structure within time window lengths as short as 30 seconds. Homotopic GCaMP6 functional connectivity maps at 0.4–4.0Hz in the anesthetized states show a striking correlated and anti-correlated structure along the anterior to posterior axis. This structure is potentially explained in part by observed propagation of delta-band activity from frontal somatomotor regions to visuoparietal areas. During awake imaging, this spatio-temporal quality is altered, and a more complex and detailed functional connectivity structure is observed. The combined calcium/hemoglobin imaging technique described here will enable the dissociation of changes in ionic and hemodynamic functional structure and neurovascular coupling and provide a framework for subsequent studies of neurological disease such as stroke.

## Introduction

Functional magnetic resonance imaging (fMRI) has been instrumental in unlocking the functional architecture of the brain and is used routinely to map patterns of resting-state functional connectivity brain networks in humans [1]. However, compared to neural activity, hemodynamic measures of brain activity (e.g. the fMRI blood oxygen level dependent (BOLD) signal) are relatively indirect and slow (<0.5 Hz). Recently developed optical approaches for mapping resting-state functional connectivity in mice, including optical intrinsic signal imaging (fcOIS), also image hemodynamics and have similar limitations [1, 2]. Functional connectivity has been used to show that functionally-related areas can have correlated spontaneous neural and hemodynamic activity, even in the absence of tasks. Similar to human fcMRI, mouse fcOIS provides a sensitive assay for several neurological diseases, including ischemic stroke [3], Alzheimer’s disease [4, 5], and optic neuritis [6] using functional connectivity. At infraslow (<0.1 Hz) and intermediate (0.08–0.4 Hz) frequencies used for most functional connectivity mapping, the hemodynamic signals are a reasonable report of neural activity [7–11]. However, as functional connectivity becomes increasingly implemented as a tool to study brain development and disease, the need for faster and more direct neuroimaging contrasts will grow. With the advent of genetic engineering techniques in mice, there are new opportunities for extending wide-field optical imaging to calcium dynamics and offer a more direct and faster measure of neural activity. Specifically, calcium concentration changes due to voltage-gated calcium channels can be imaged and visualized using fluorescent, genetically encoded calcium indicators (GECIs) [12]. These fluorophores, including GCaMP [13], are generated from a modified green fluorescent protein coupled to a calcium binding domain, enabling increased fluorescence in the presence of elevated calcium levels. GECI’s have been used extensively to study *in vivo* neural activity in *C*. *elegans* [13], mice [8, 13–17], rats [18], monkeys [19], and other animal models.

GECIs also offer the opportunity to map spontaneous network activity at frequencies >0.5 Hz. By opening up access to higher frequencies, GECIs can also be used to observe phenomena that are not accessible with hemodynamics such as the delta-band slow oscillation (0.5–4.0Hz) present in sleep and under anesthesia [20]. The delta-band slow wave sleep phenomenon, most often observed and reported using electrophysiological techniques [21–23], consists of synchronous, oscillating alternations between depolarized and hyperpolarized neuronal states. As these waves of delta-band activity propagate over the cortex, they may interact with underlying spontaneous activity stereotypical of functional connectivity structure. For example, in humans, a fragmentation of electrophysiological functional connectivity and an increase in ~1Hz cortical activity has been observed following propofol-induced loss of consciousness [24].

In mice, GECIs provide a unique approach for examining these oscillations and their relation to spontaneous functional connectivity structures. Though electrophysiological methods can provide improved temporal sensitivity to synaptic transmission than fluorescent calcium sensors, they present many challenges to practically implement systems that provide extended spatial information within and across functional networks [25]. The similarities of *in vivo* imaging protocols (e.g. illumination sources, detection) between fluorescent calcium imaging and OIS imaging lend to more feasible implementation of multimodal, concurrent acquisition compared to an analogous combined electroencephalography (EEG)-intrinsic signal system. Combined with the ability to target GECI expression to investigate activity from specific cell populations and types, GCaMP6 is a compelling instrument in the understanding of functional neurophysiology beyond the lens allowed by hemodynamic-based imaging methods.

The goal of this study is to establish the state- and frequency-dependence in functional connectivity and delay topographies in spontaneous cortical calcium transients. In this paper, we report concurrent imaging of hemoglobin and GCaMP6 dynamics in a *Thy1*/GCaMP6 transgenic mouse model [17]. An optical imaging system and algorithms were developed to image wide-field calcium and hemoglobin dynamics concurrently over three decades of frequency (0.008Hz to 8 Hz). We first quantified the duration of spontaneous GCaMP6 data necessary to construct canonical functional connectivity images. The patterns of functional connectivity within the infraslow (0.008–0.09Hz) spontaneous GCaMP6 and HbO_2_ dynamics were then evaluated using canonical seed-based intercontrast functional connectivity analysis. Since GCaMP6 enables analysis at higher frequencies, we characterized functional connectivity maps within delta-band (0.4–4.0Hz) GCaMP6 activity across the cortex in both anesthetized and awake states. Within the higher-frequency delta band, we evaluate the speed with which the connectivity analysis stabilized. We also evaluated the possibility of a state-dependence of calcium-based functional connectivity topography. Spectral features of GCaMP6 dynamics were compared to simultaneously-measured EEG to validate the sensitivity of GCaMP6 to global cortical electrophysiological activity and to state (awake or anesthetized). Finally, cortical propagation in GCaMP6 was examined during the transition from anesthetized state to wakefulness. The method for concurrent GCaMP6/hemodynamic imaging developed herein will provide a framework both for mapping calcium- and hemoglobin-based functional connectivity and improving our understanding of spontaneous network activity.

## Methods

### *Thy1*/GCaMP6 transgenic mice

Transgenic mice expressing GCaMP6 driven by the thymus cell antigen 1 (*Thy1*) promotor were acquired from Jackson Laboratories (JAX Strain: C57BL/6J-*Tg(Thy1-GCaMP6f)GP5*.*5Dkim*; stock: 024276)[17, 26]. In this model, GCaMP6 expression is widespread in the CNS (e.g. cortex, hippocampus, thalamus, and cerebellum) and localized predominantly to excitatory projection neurons [27]. *Thy1*/GCaMP6 transgenic genotypes were confirmed by PCR using the forward primer 5’-CATCAGTGCAGCAGAGCTTC-3’ and reverse primer 5’- CAGCGTATCCACATAGCGTA-3’.

### Animal preparation

All procedures have been approved by the Washington University School of Medicine Animal Studies Committee. Seven GCaMP6 mice (12-16weeks of age, 28-36g) were used in all experiments in this study. Mice were sedated with isoflurane (3% induction, 1% maintenance, 0.5L/min) and placed in a stereotactic holder. The head was then shaved, and a midline incision made to expose the skull. Body temperature was maintained at 37°C using a temperature controlled heating pad. Following scalp retraction, pilot holes were drilled into but not penetrating the skull at approximately -1mm posterior to bregma, and +/- 5mm lateral to bregma (near barrel/auditory cortex). Small stainless steel self-tapping screws (BASI Inc., West Lafayette, IN, USA) were then inserted into these holes for collecting EEG recordings. Chronic cranial windows made of Plexiglas and with pre-tapped holes were fixed to the skull using dental cement (C&B-Metabond, Parkell Inc., Edgewood, NY, USA).

### *Ex vivo* epifluorescence and confocal imaging

Mice were deeply anesthetized with FatalPlus^TM^ (Vortech Pharmaceuticals, Dearborn, MI, USA) and transcardially perfused with 0.01M PBS. The brains were removed and fixed in 4% paraformaldehyde for 24h and transferred to 30% sucrose in 0.2M PBS. After brains were saturated, they were snap-frozen on dry ice and coronal sections, 50μm thick, were made with a sliding microtome. Sections were stored in 0.2M PBS, 30% sucrose, and 30% ethylene glycol at −20°C. For viewing, cut sections were washed in PBS, mounted, and intrinsic GCaMP6 fluorescence was examined with epifluorescence microscopy (Nikon Eclipse 80i, Nikon Instruments Inc., Melville, NY, USA). For viewing using confocal microscopy, additional cut sections were washed in PBS, mounted, and coverslipped in DAPI containing mounting media (Vector Laboratories, Burlingame, CA, USA). Fluorescent images of DAPI labeled cells and intrinsic GCaMP6 fluorescence were then acquired with a Nikon A1-Rsi inverted confocal microscope. Excitation illumination was provided by 405nm and 488nm lasers for DAPI and GCaMP fluorescence, respectively (Nikon Eclipse 80i, Nikon Instruments Inc., Melville, NY, USA).

### *In vivo* optical imaging system

Sequential illumination was provided by four LEDs: 470nm (measured peak λ = 454nm, LCS-0470-15-22, Mightex Systems, Pleasanton, CA, USA), 530nm (measured peak λ = 523nm, LCS-0530-15-22), 590nm (measured peak λ = 595nm, LCS-0590-10-22), and 625nm (measured peak λ = 640nm, LCS-0625-03-22). All LEDs are referred to by their measured wavelengths (i.e. 454nm, 523nm, 595nm, and 640nm) throughout the rest of the manuscript. The 454nm LED is used for GCaMP excitation, and the 523nm, 595nm, and 640nm LEDs are used for multispectral oximetric imaging. The 523nm LED was also used as an emission reference for GCaMP6 fluorescence in order to remove any confound of hemodynamics in the fluorescence signal (described below). Both the 454nm and 523nm LED light paths were made collinear by using a multi-wavelength beam combiner dichroic mirror (LCS-BC25-0505, Mightex Systems, Pleasanton, CA, USA). For image detection, we used a cooled, frame-transfer EMCCD camera (iXon 897, Andor Technologies, Belfast, Northern Ireland, United Kingdom) in combination with an 85mm f/1.4 camera lens (Rokinon, New York, NY, USA). The acquisition framerate was 16.8Hz per channel, with the overall framerate of the camera as ~67Hz. This framerate is well above respiration rates (approximately 3Hz) and fast enough to adequately characterize GCaMP6 activity. To increase frame rate as well as increase SNR, the CCD was binned at 4 x 4 pixels; this reduced the resolution of the output images from full-frame 512 x 512 pixels to 128 x 128 pixels but allowed for increased frame rate. Both the LEDs and the exposure of the CCD were synchronized and triggered via a DAQ (PCI-6733, National Instruments, Austin, TX, USA) using MATLAB (MathWorks, Natick, MA, USA). The field-of-view was adjusted to cover the majority of the convexity of the cerebral cortex with anterior-posterior coverage from the olfactory bulb to the superior colliculus, resulting in an area of approximately 1cm^2^ with each pixel being approximately 78μm x 78μm. To minimize specular reflection from the skull, we used a series of linear polarizers in front of the LED sources and the CCD lens. The secured mouse was placed at the focal plane of the camera. The combined, collimated LED unit was placed approximately 8cm from the mouse skull, with a working distance of approximately 14cm as determined by the acquisition lens. A 515nm longpass filter (Semrock, Rochester, NY, USA) was placed in front of the CCD to filter out 470nm fluorescence excitation light and a 460/60nm bandpass filter (Semrock, Rochester, NY, USA) was used in front of the excitation source to further minimize leakage of fluorescence excitation light through the 515nm longpass filter. The pulse durations for the LEDs are 20ms, 5ms, 3ms, 1ms for 454nm, 523nm, 595nm, and 640nm, respectively. All imaging data from awake and anesthetized imaging sessions were acquired and stored in separate 5min imaging runs.

### Electrical hindpaw stimulation

Electrical stimulation was generated using an isolated pulse stimulator (Model 2100, A-M Systems, Carlsborg, WA, USA) delivered to the left hindpaw of each mouse via micro vascular clips (Roboz Surgical Instrument Co., Gaithersburg, MD, USA). The somatosensory activation block paradigm consisted of an initial rest period of 5s followed by 4s of electrical stimulation at a frequency of 2Hz (pulse duration: 300μs, current: 0.5mA) and ending with 51s of rest. This 60s block was repeated five times during each 5min imaging session for subsequent block averaging.

### Anesthetized imaging

For anesthetic imaging, mice were anesthetized with intraperitoneal injection of a ketamine/xylazine cocktail (86.9mg/kg ketamine, 13.4mg/kg xylazine). Anesthetic effect was verified by confirming that the animal was not responsive to a hind paw pinch. The animal was placed and kept on a solid state water circulating heating pad (T/Pump Classic, Stryker Co., Kalamazoo, MI, USA), maintained at 42°C, to minimize electronic noise in the EEG signal.

### Awake imaging

We constructed a customized apparatus consisting of a felt pouch suspended by optical posts. This provided a dark, comfortable environment for the mouse throughout data acquisition. During the imaging session, the mouse was able to move freely while its head was secured, preventing the awake mouse from applying torque on their restrained head and optical window. Though no accelerometers or other behavioral measures were used to track motion within the pouch during imaging, after acclimation, mice are typically relaxed with infrequent limb motion. After recovery from this surgery (1 week), the mouse was acclimated to the apparatus by a training period consisting of two 20min sessions. Acclimation is indexed by a return to normal behavior (e.g., whisking, grooming, and walking with head restrained). Once acclimated, mice were mounted to the imaging system with a small bracket (using the window’s pre-tapped holes) with the body of the mouse placed inside the felt pouch. Imaging then proceeded as described above.

### EEG recording and processing

Summed field potentials were concurrently recorded during GCaMP6 and OIS imaging using the 2 screws bilaterally implanted in the skull and subsequently digitized at 1kHz (Power Lab EEG Amplifier, AD Instruments, Dunedin, New Zealand). EEG frequency spectra were calculated by splitting data from each 5min session into thirty 10s epochs, applying a 10s Hann window to each, calculating the Fast Fourier Transform, then averaging the squared modulus of each epoch together.

### Image processing

A representative frame of baseline light levels in a dark environment, calculated from a mean of dark images collected over 1min, was subtracted from the raw data. All pixel time traces were detrended to remove any variations in light levels due to photobleaching, LED current drift, and nonuniformity across the skull [28]. Reflectance changes in the 523nm, 595nm, and 640nm LED channels were used in combination to provide oximetric data using the Modified Beer-Lambert Law, described previously [2]. Images in each contrast were smoothed with a Gaussian filter (5x5 pixel box with a 1.3 pixel standard deviation).

The GCaMP6 fluorescent signal must be corrected for any contribution from the vascular compartment and varying concentrations of absorptive hemoglobin. Common correction methods to calculate relative fluorescence changes include using a reference wavelength for applying subtraction and ratiometric techniques. We implemented a ratiometric correction algorithm (Eq 1) to correct fluorescent emission for any absorption by hemoglobin and deoxyhemoglobin using the reflectance channels at the GCaMP6 emission wavelengths (523nm LED) as a reference.

$$y(t)=\frac{I^{em}(t)}{I^{ref}(t)}\cdot \frac{I_{0}^{ref}}{I_{0}^{em}}$$ (1)

*y(t)* is the final corrected GCaMP6 time series for a given pixel, *I*^*em*^ refers to the detected fluorescent emission intensity. *I*^*ref*^ describes the measured reflectance changes at the emission wavelength. A single frame from the 628nm reflectance channel was loaded into Adobe Photoshop CC 2014 (Adobe Systems, San Jose, CA, USA) and all regions not corresponding to brain were manually painted white. The image was loaded back into MATLAB and used to create a binary brain mask. All subsequent analysis was performed on those pixels labeled as brain. Image sequences from each mouse (as well as the brain mask for each mouse) were affine-transformed to a common atlas space (based on the Paxinos mouse atlas) using the positions of bregma and lambda[29]; please see S3 Fig for cortical region parcels corresponding to the network seeds used in analysis and White and Bauer et al., for more information [2]. The time traces of all pixels defined as brain by the binary mask were averaged to create a global brain signal. For all functional connectivity and activation analyses, this global signal was regressed from every pixel’s time trace to remove global sources of variance, such as motion and cardiac pulsations, thus increasing correlation network specificity [2, 30, 31]. For all whole-brain delay analyses, the global brain signal was not regressed from the spontaneous data, as it served as the temporal point of reference for the cross-correlation calculations. All resting-state data were filtered over the canonical functional connectivity frequency range (0.009–0.08Hz) [1], an intermediate frequency band (0.08–0.4Hz), or the delta activity band (0.4–4.0Hz). Evoked activity was lowpass filtered at 6Hz to remove high-frequency noise.

### Spectral analysis

To construct frequency spectra of spontaneous GCaMP6 activity, time series at each pixel were split into two 150s windows (per each 5min imaging session), windowed with 150s long Hann functions, and then the Fast Fourier Transform (FFT) was calculated. The squared moduli of these FFTs were then averaged across the brain to produce the final power spectra.

### Seed-based functional connectivity

Using the Paxinos histological atlas as a reference [29], seed locations were chosen at coordinates corresponding to the left and right visual, motor, somatosensory, cingulate, parietal, auditory, and retrosplenial cortices as well as the right and left superior colliculi and olfactory bulbs. A 0.5mm diameter circle at each seed location was averaged to create a seed time trace. These seed traces were correlated against every other brain pixel using the Pearson correlation coefficient (*r*) to create functional connectivity maps. Functional connectivity maps were calculated for every 5min imaging session, Fisher z-transformed (using the inverse hyperbolic tangent function) to improve normality and stabilize the variance of the correlation coefficients, averaged across all imaging sessions and mice, then finally reverse transformed (using the hyperbolic tangent function) back to correlation coefficients. Matrices consisting of the correlation coefficient between each pair of network seed traces were constructed by plotting the pairwise correlation coefficients in each cell. Dice’s coefficient was adapted (Eq 2) to compare spatial similarity of variable length time and sampling window functional connectivity maps and maps created using group averaged. The adapted Dice’s coefficient was calculated using:

$$s=\frac{2\sum_{i=1}^{d}A_{i}B_{i}}{\sum_{i=1}^{d}A_{i}^{2}+\sum_{i=1}^{d}B_{i}^{2}}$$ (2)

Where *s* is the spatial similarity, *A* and *B* are the functional connectivity maps being compared, an *i* indexes each pixel within the maps.

### Whole-brain delay analysis

The GCaMP6 time series from every pixel was cross-correlated with the global signal (described above) to reveal any phase shifts (quantified as time lags). The temporal shift that maximizes the correlation between both time series was plotted at each pixel to create a map of delay topography across the cortex.

### Statistical analysis

To compare differences in functional connectivity network maps across the anesthetized and awake states, two-tailed Welch’s unequal variances t-tests were used to compare Fisher z-transformed correlation coefficients at every pixel. T-statistic maps were then created and subsequently thresholded using a Bonferroni-adjusted significance level of α = 3.1e-6, calculated by adjusting a conventional α = 0.05 significance level by the total number of pixel comparisons (128 pixels x 128 pixels, 16384 pixel comparisons in total). Two-tailed paired t-tests were used to compare differences at each pixel in the visual network functional connectivity maps in the mouse transitioning from anesthesia to wakefulness. Each 5min epoch was divided into ten 30s windows for all comparisons across epochs. Calculated t-statistic maps were thresholded using q = 0.001 after false discovery rate adjustment. Two-tailed Welch’s unequal variances t-tests were used to compare the full width at half maximum values for both the ipsilateral and contralateral horizontal line profiles from each seed-based functional connectivity map.

## Results

### Concurrent imaging of GCaMP6 fluorescence and hemodynamics

**Fig 1:**
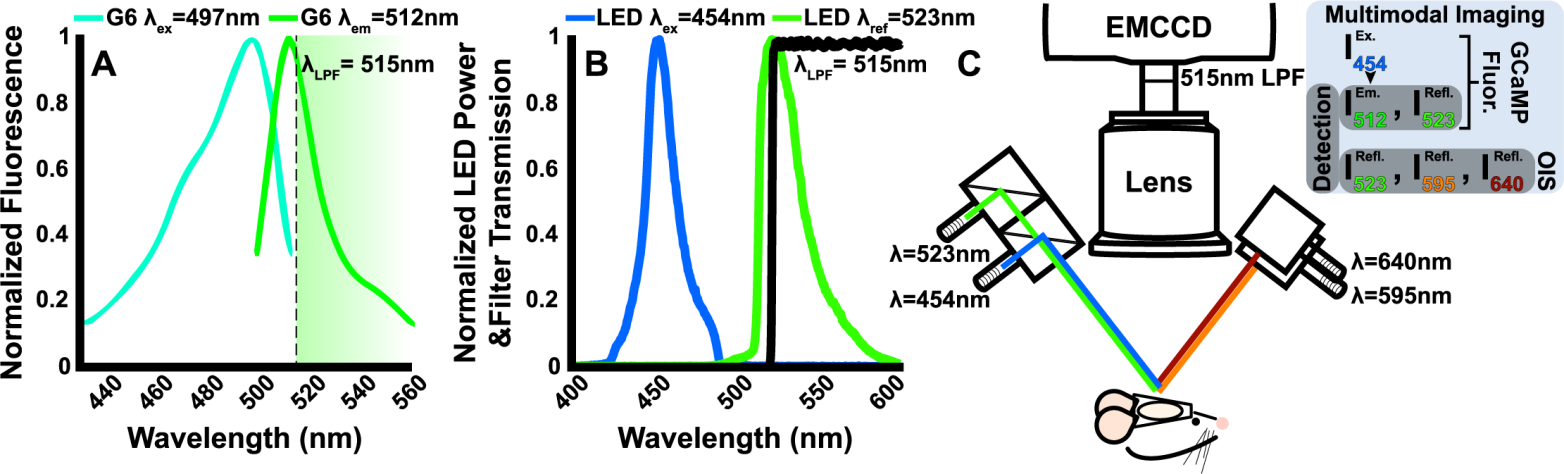
Concurrent GCaMP6 fluorescence and optical intrinsic signal imaging system. LED wavelengths must be selected to provide GCaMP6 excitation, distinguish between oxy- and deoxyhemoglobin absorption for oximetry, and enable correction of GCAMP6 fluorescence emission for correction of confounding hemoglobin absorption. (A) GCaMP6 dynamics are imaged using fluorescence measurements, wherein the fluorophore has a peak excitation at λ = 497nm and peak emission at λ = 512nm. A longpass filter at λ = 514nm is used to block fluorescence excitation light. (B) Spectra from the excitation LED (λ = 454nm, bandpass filtered at 460/60nm). A reference (λ = 523nm) LED is used for correcting for changes in optical properties due to the presence of absorptive hemoglobin. The transmission curve for the 514nm longpass filter at detection is shown for reference. (C) Schematic of the combined GCaMP and OIS imaging system. GCaMP excitation and reference LEDs share a collinear optical path using dichroic mirrors. Green (λ = 523nm), yellow (λ = 595nm), and red (λ = 640nm) are used together to determine differential concentration changes of hemoglobin. Detection is done using a cooled EMCCD with an 85mm f/1.4 lens.

In order to image both GCaMP6 activity and hemodynamics concurrently, we developed an optical imaging system with multiple illumination wavelengths that were temporally multiplexed. A filtered detection scheme provided both fluorescence and reflectance imaging. To image GCaMP6 fluorescence, we selected incident LEDs wavelengths that match both the excitation (peak lambda = 497nm) and emission (peak λ = 512nm) spectra of GCaMP6 (Fig 1A and 1B). To correct the raw fluorescence measurement for hemodynamic absorption changes, we incorporated a reference reflectance measure (green reflectance measurement using a LED with peak λ = 523nm) in a ratiometric correction technique (see methods for details). The green LED was also used along with yellow (peak λ = 595nm) and red (peak λ = 640nm) LEDs for concurrent calculation of differential changes of hemoglobin concentration (see Methods) (Fig 1C). The excitation light (peak λ = 454nm) was blocked between the lens and the detector using a 515nm longpass filter (Fig 1A–1C. Light was detected for all wavelengths using an electron multiplying charge coupled detector (EMCCD, iXon 897, Andor Technologies). Sequentially triggering each of the four LEDs yielded GCaMP6 and hemodynamic frame rate of16.8Hz.

### GCaMP6 fluorescence is detectable and temporally distinct from HbO_2_

**Fig 2:**
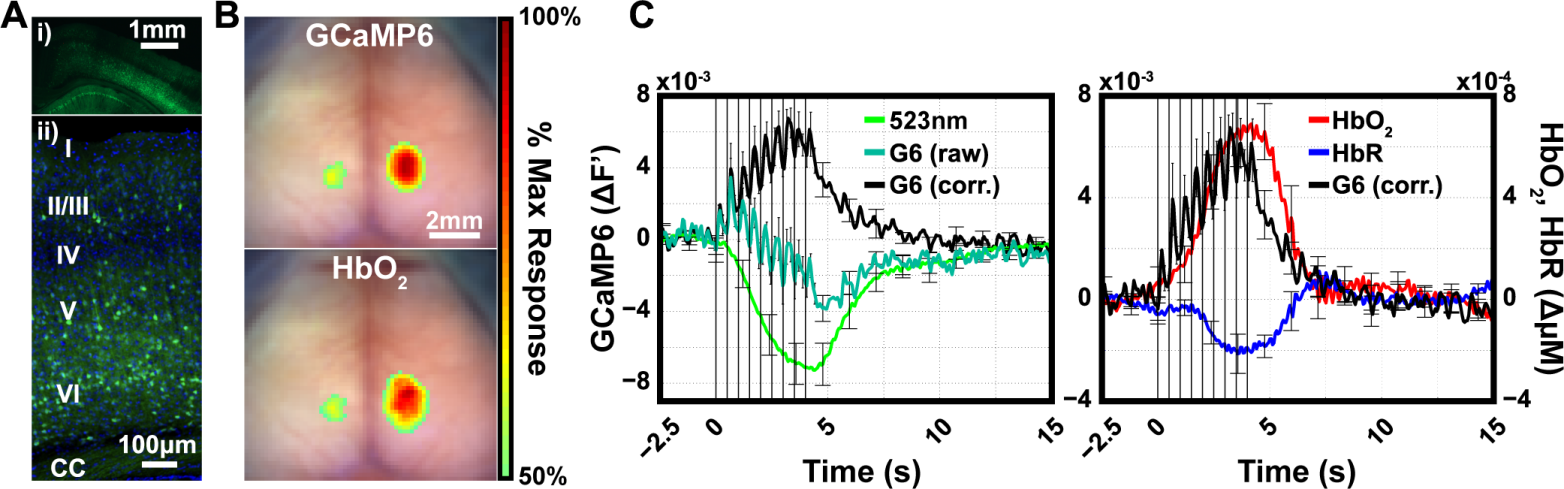
*Thy1*/GCaMP6 mouse model validation. Based on previous studies, *Thy1*/GCaMP6 fluorescence should provide a signal predominantly driven by cortical neuronal neurons and that is faster than hemodynamic measures. (A) Epifluorescence (i) and confocal (ii) images of 50um coronal slices collected from a representative *Thy1*/GCaMP6 mice. GCaMP6 expression (green) is located throughout the cortex (i) and localized to neuronal soma (ii, blue-DAPI stain) and can be seen throughout cortical layers I-VI. (B) Map of stimulus evoked responses to electrical stimulation of left hindpaw (thresholded at >50% peak response) in the anesthetized state. (C) Time series of evoked responses sampled using masks created from the right half of the 50% peak response images in part B. Green reflectance (green trace, top panel) is used to correct raw GCaMP6 emission (teal trace) for changes in absorption due to hemodynamics using a ratiometric approach (see Methods; the corrected trace is black). The corrected GCaMP6 trace (black) is sensitive to each stimulus presentation (black trace, lower panel), whereas HbO_2_ (red) and HbR (blue) follow the slower, canonical hemodynamic response. Error bars are standard error of the mean. Data from 36 blocks across 7 mice are included in all evoked analysis.

To confirm GCaMP6 fluorescence was present in superficial cortical layers, paraformaldehyde-fixed GCaMP6 brain slices were imaged using both epifluorescence (Fig 2Ai) and confocal (Fig 2Aii) microscopy. Individual neuronal somas (Fig 2Aii, green: GCaMP6, blue: DAPI stain) and their neurites are detectable throughout cortical layers I–VI (Fig 2A) and across the cortex (Fig 2Ai, green: GCaMP6).

To evaluate the mapping fidelity of both the GCaMP6 and hemoglobin contrasts and the ratiometric correction strategy, we used a block design electrical hindpaw stimulation paradigm (each block consisted of 4s of stimulation at 2Hz, followed by 51s of rest; 36 blocks across n = 7 mice were included in analysis). Mice were under ketamine/xylazine anesthesia during electrical stimulation presentations to minimize discomfort and distress. Maps of the spatial extent of hindpaw cortex, contralateral to stimulation, for both GCaMP6 and HbO_2_ show well localized responses (Fig 2B). Before ratiometric correction, each electrical pulse response is represented in the raw GCaMP6 signal (Fig 2C, dark green). However, during the stimulus, after initially rising, the raw fluorescence signal rapidly decreases, owing to increased absorption (Fig 2C, light green) from the increased blood volume and hemoglobin concentration (Fig 2C, red trace). After ratiometric correction for absorption, the GCaMP6 signal increases and stays elevated until returning to baseline approximate 6s post-stimulus. The evoked responses corresponding to HbO_2_ (Fig 2C, red trace) and HbR (Fig 2C, blue trace) have an onset approximately 2s after the initial stimulus presentation. Also, the observed ratio of HbO_2_ to HbR peak magnitudes is approximately 3:1, as expected from canonical functional responses [32]. These results collectively demonstrate the ability of the hardware and algorithms to concurrently image both calcium dynamics (via GCaMP6) and hemodynamics.

### Functional connectivity mapping in GCaMP6 mice

**Fig 3:**
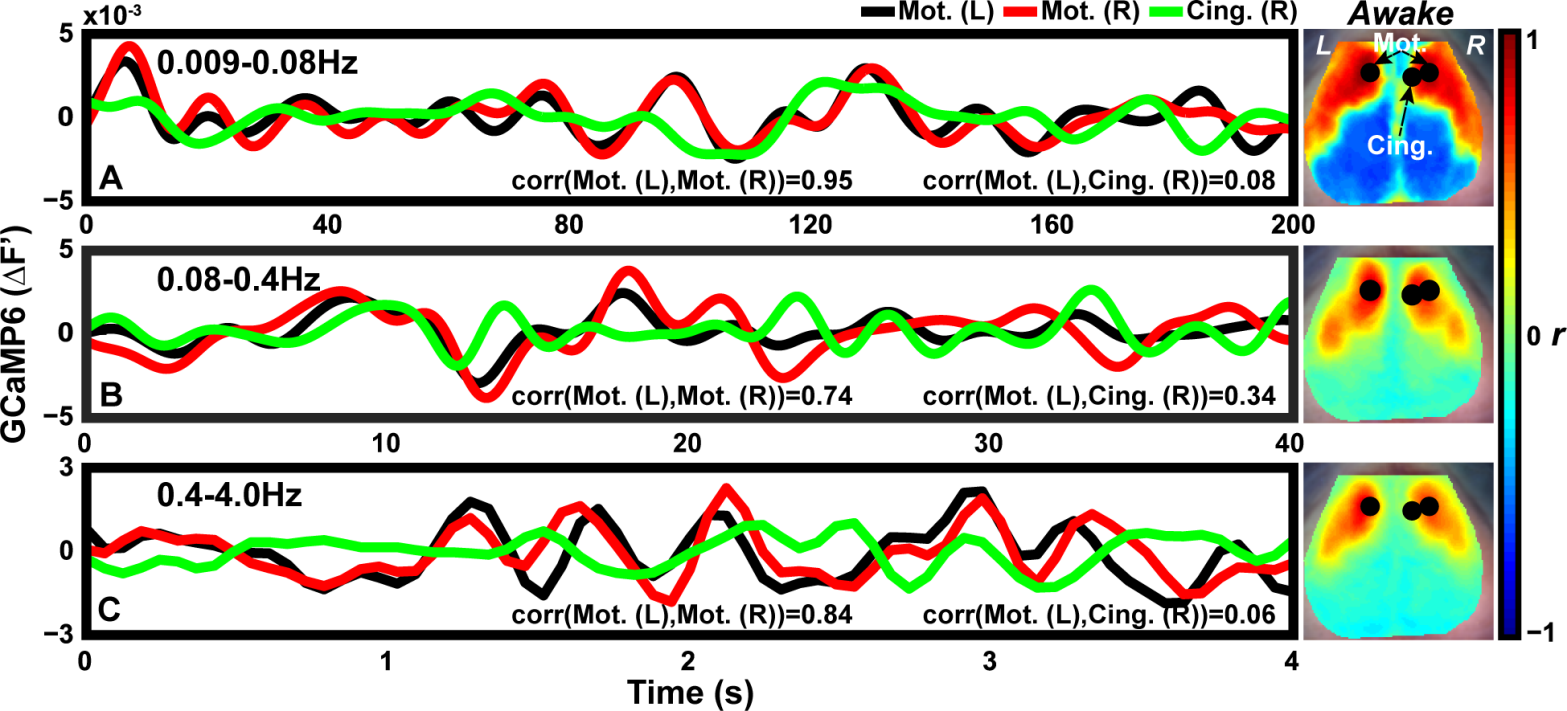
Performing functional connectivity analysis in the awake mouse brain. Time series of spontaneous GCaMP6 activity filtered over the canonical functional connectivity band: (A) 0.009–0.08H, (B) 0.08–0.4Hz, and (C) the delta band: 0.4–4.0Hz. Corresponding functional connectivity maps constructed using a seed from left motor (Mot.) cortex (black circle) in 0.009–0.08Hz (top right), 0.08–0.4Hz (center right), and 0.4–4.0Hz (bottom right) spontaneous data are also shown. Black and red traces correspond to left and right motor cortices, respectively. The green trace is sampled from right cingulate cortex. Over both frequency bands, left and right motor cortex show highly correlated activity whereas right motor with left cingulate show reduced temporal correlation. At the pixelwise level, correlating the time series from the left motor (black) with every pixel in the brain produces a map showing homotopic functional somatosensory structure across hemispheres. Mot.: Motor, Cing.: Cingulate.

Functional connectivity was quantified by calculating the correlation between the time traces in two distinct brain regions (Fig 3). To facilitate comparisons of fcMRI and the functional connectivity analysis using the concurrent GCaMP6/hemodynamic imaging system, we implemented a standard functional connectivity processing pipeline across three frequency bands: 0.009–0.08Hz (“infraslow”), 0.08–0.4Hz (“intermediate”), and 0.4–4.0Hz (“delta-band”) for all contrasts. Seed-based functional connectivity was then performed using spontaneous GCaMP6 and HbO_2_ activity across frequency bands and awake and anesthetized states. In an illustrative example of spontaneous GCaMP6 data measured in wakefulness, the time series sampled from left motor and contralateral right motor cortices (Fig 3, black & red traces and circles, respectively) over the infraslow band are highly correlated (r = 0.95), whereas the correlation between the left motor seed and right cingulate cortices is low (r = 0.08). Functional connectivity maps were constructed by calculating the position-dependent correlation coefficient between this left motor seed and the spontaneous time series at every pixel across the field-of-view (Fig 3A, right; representative time series from all contrasts and frequency bands, S4 Fig). A recent wide-field *in vivo* optical imaging study of *Thy1*/GCaMP6 mice by Ma and colleagues showed that spontaneous GCaMP6f and hemodynamic activity from awake mice were most correlated when filtered with a 0.35Hz-wide frequency-domain window centered at 0.21Hz [33]. Based on this observation, resting-state data were also filtered over an intermediate frequency band (0.08–0.4Hz) to facilitate exploration of GCaMP6f and hemodynamic correlated activity. Fig 3B shows representative spontaneous seed traces from Motor and Cingulate networks from this 0.08–0.4Hz band. This same analysis was applied to the higher frequency delta band (Fig 3C).

**Fig 4:**
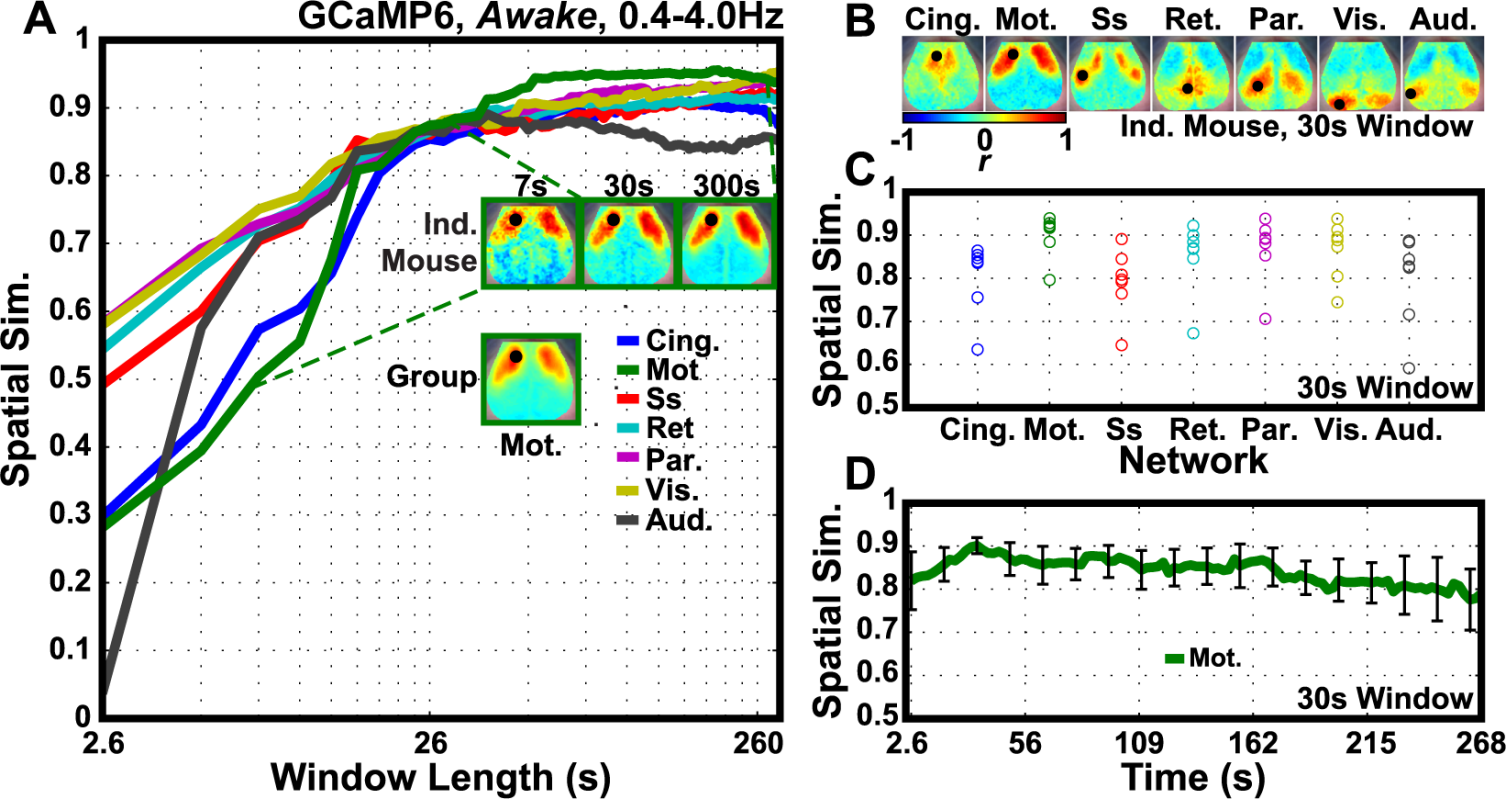
Stability and stationarity of spontaneous GCaMP6 activity in functional connectivity analysis. (A) Functional connectivity maps were constructed using time windows of spontaneous 0.4–4.0Hz GCaMP6 (awake) data of increasing length from one mouse (Mouse 2). Windows were increased in length by ~2.6s up to ~300s. Spatial similarity (Dice coefficient) was calculated between each of these functional connectivity maps for all networks relative to their corresponding group level map. The functional connectivity maps calculated by using a seed in left motor cortex are highlighted as an example. Across all networks, similarity scores converge at 0.9 with a window length of ~30s. (B) Seed-based functional connectivity maps from Mouse 2 constructed using 30s of spontaneous GCaMP6 data. (C) For each of 7 networks, the same 30s window of spontaneous data for each network was compared to the corresponding group-level network functional connectivity map for each of seven mice (circles). The stability of similarity of 30s of data is present across network and across mice. (D) Using consecutive 30s windows (sampled every ~2.6s) from a single 300s imaging session from each mouse, the spatial similarity between all 30s motor functional connectivity maps and the group level motor functional connectivity map is shown to be relatively stationary. Error bars are standard error of the mean.

Because GCaMP6 is able to probe activity at frequencies higher than traditionally associated with hemodynamic functional connectivity (i.e. <0.1Hz), we examined the effect of time window length on the stability of the functional connectivity patterns using the highest frequency GCaMP6 data examined in this study which are filtered over the delta frequency band (0.4–4.0Hz). To avoid confounds from the effects of anesthesia (e.g. ketamine-associated increase in cortical activity in this delta frequency range), awake data were used [21]. A spatial similarity analysis was employed to evaluate the convergence of network structures with increasing duration of resting-state data. For each window length (from 2.6s to 300s in duration) and for each seed location, we calculated the similarity between representative individual mouse data and a respective reference template calculated from longer data acquisitions with averaging across all mouse (i.e. group-level) maps (Fig 4A). The same analysis was carried out using the wakefulness data filtered over the infraslow frequency band (S5 Fig). In contrast to Fig 4A, the window length necessary to reach convergence is more network-dependent and most networks do not achieve a spatial similarity score of at least 0.5 compared to the group average reference with window lengths shorter than 100 seconds. Using window lengths as short as 30s for data from an individual mouse, the functional connectivity maps constructed with 7 canonical seeds exhibited distinct qualitative network-specific structure (Fig 4B). The similarity between functional connectivity maps based on 30s of data vs. the group is consistent for all 7 networks for all mice (Fig 4C). To evaluate the stability of the maps over time, the functional connectivity map for a seed placed in motor cortex was constructed for a sequence of overlapping 30s segments of data, shifted by increments of 2.6 seconds, for all mice and compared to the group-averaged map. The similarity score remains relatively consistent across the entire 5min session (Fig 4D). Collectively, these data imply that windows with a duration as short as 30s can produce stable functional connectivity maps when using GCaMP6 data. These results show that the robustness of GCaMP6 functional connectivity maps from these 30s windows is relatively independent of temporal sampling location, network seed, and mouse.

### Intercontrast functional connectivity between delay-shifted GCaMP6 and HbO_2_ recapitulates hemodynamic functional connectivity structure in the anesthetized state

**Fig 5:**
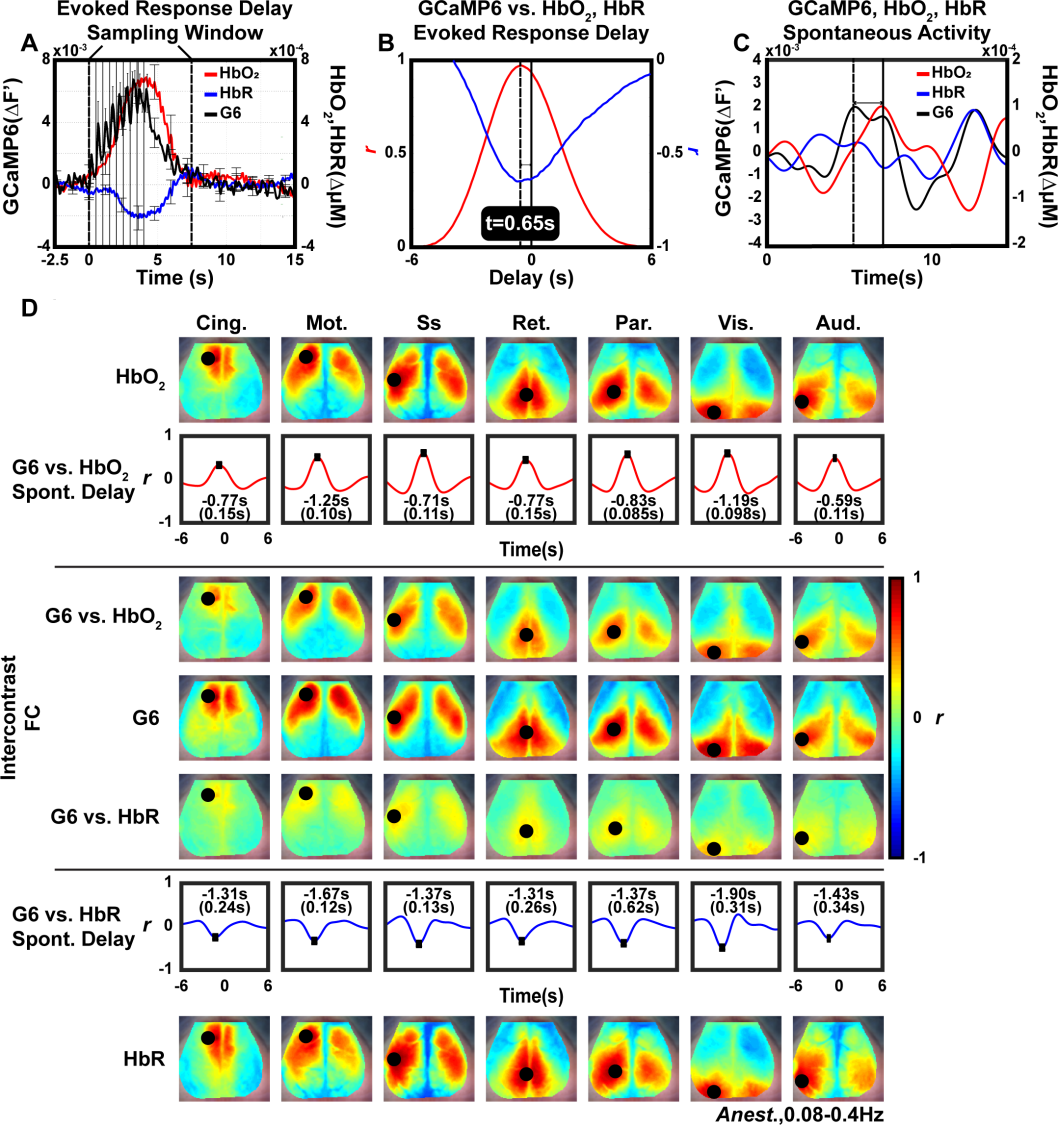
Intercontrast functional connectivity mapping in the anesthetized state. Based on the temporal delay between evoked GCaMP6 and HbO_2_/HbR responses combined with similar observed 0.08–0.4Hz functional connectivity structure, delay-shifted GCaMP6 likely shares functional network information with HbO_2_ and HbR. (A) GCaMP6 (black), HbO_2_ (red), and HbR (blue) evoked responses to 2Hz 0.5mA electrical stimulation of left hind paw (see Fig 2B and 2C). Data from a sampling window spanning from approximately t = 0s to t = 7.5s were included in subsequent cross-correlational delay analysis. (B) Cross-correlation from the GCaMP6 and HbO_2_ time courses in (A), and the GCaMP6 and HbR time courses in (A), both show a delay of 0.65s between responses. (C) An example of spontaneous GCaMP6, HbO_2_, and HbR time traces from the right motor cortex seed over the 0.08–0.4Hz band. HbO_2_ and HbR data was shifted based on the lag with the maximum correlation calculated between the spontaneous hemoglobin data and spontaneous GCaMP6 data per seed (as shown by arrows). (D) Top and Bottom: Seed-based group-averaged functional connectivity mapping in filtered (0.08–0.4Hz) anesthetized data using HbO_2_ (top) or HBr (bottom) and cross-correlation curves (with mean delays of maximum correlation in black text with standard errors of the mean in parenthesis) between each HbO_2_ network seed trace and corresponding GCaMP6 seed trace. Middle: Seed-based GCaMP6 functional connectivity maps and corresponding intercontrast seed-based functional connectivity maps for GCaMP6 vs temporally-shifted HbO_2_ (top) and GCaMP6 vs temporally-shifted HbR (bottom). Correlation between spontaneous HbO_2_ and GCaMP6 contrasts (filtered between 0.08–0.4Hz) are better optimized by removing the intercontrast time shift between these contrasts compared to the analogous correlation between spontaneous HbR and GCaMP6 contrasts.

Though the origins of the GCaMP6 and HbO_2_ signals are distinct, they are associated via neurovascular coupling [34, 35]. Accordingly, we examined the potential interaction between 0.08–0.4Hz GCaMP6 and HbO_2_ dynamics. This intermediate frequency band was selected given the maximal correlation recently observed between spontaneous GCaMP6 and HbT at approximately 0.21Hz [33]. Data from the anesthetized state were selected to directly build upon previously-reported literature on functional connectivity using OIS hemodynamic contrasts [2–6, 36–38]. Further, the delay times derived from spontaneous data could be compared to the delay time from forepaw stimulation. By calculating the lagged-correlation between the GCaMP6 and HbO_2_ evoked responses to electrical hindpaw stimulation (Fig 2C), we measured the temporal delay of HbO_2_ relative to GCaMP6 to be ~0.65s (Fig 5A and 5B), which is comparable in magnitude to the previously reported delay of maximal correlation calculated between spontaneous GCaMP6 and HbT (~0.86s) in this frequency band [33]. Due to the lack of full characterization of the relationship and neurophysiology of neurovascular coupling in evoked versus spontaneous activity conditions, we also carried out this analysis by delay-shifting spontaneous data using delays determined from seed-trace pairs of resting-state data (Fig 5C and 5D). For reference, we evaluated the same seed locations previously used for hemodynamic functional connectivity mapping in spontaneous data acquired from mice under anesthesia [2, 3, 6](S1 Fig). Within the intermediate frequency band, GCaMP6 produced intact functional connectivity patterns (Fig 5D, middle) similar to concurrent HbO_2_ maps (Fig 5D, top; S2 Table), as well as to maps made using deoxyhemoglobin (HbR) (Fig 5D, bottom; S2 Table). Next, to evaluate the potential relationship between spontaneous GCaMP6 activity and HbO_2_ and HbR dynamics, we temporally shifted spontaneous GCaMP6 traces for the seven seeds by the computed spontaneous delay between HbO_2_ (Fig 5D, top, bottom row) and HbR (Fig 5D, bottom, top row) and then calculated correlation maps between the GCaMP6 seeds and the shifted HbO_2_ and HbR data (Fig 5D, middle). The resulting maps show distinct patterns spatially similar to the within-GCaMP6 functional connectivity maps (Fig 5D middle, S3 Table). The same spontaneous delay-shift analysis was carried out on infraslow data (S6 Fig, S3 Table), producing functional connectivity maps with ill-defined network boundaries. To further investigate the relationship and neurophysiology of neurovascular coupling in evoked conditions, we also carried out the same delay-shifting analysis using the fixed calculated ~0.65s shift (S7 Fig); the results generally recapitulate those reported here (Fig 5, S3 Table). Moreover, intercontrast functional connectivity analysis between GCaMP6 and HbR produced functional connectivity maps with largely indeterminate network-specific spatial structure (Fig 5, S6 and S7 Figs, S3 Table). These results imply that both signals are synchronized, with GCaMP6 potentially containing predictive information of downstream hemodynamics.

### At higher frequencies, GCaMP6 shows a striking state-dependence in correlation map structure

**Fig 6:**
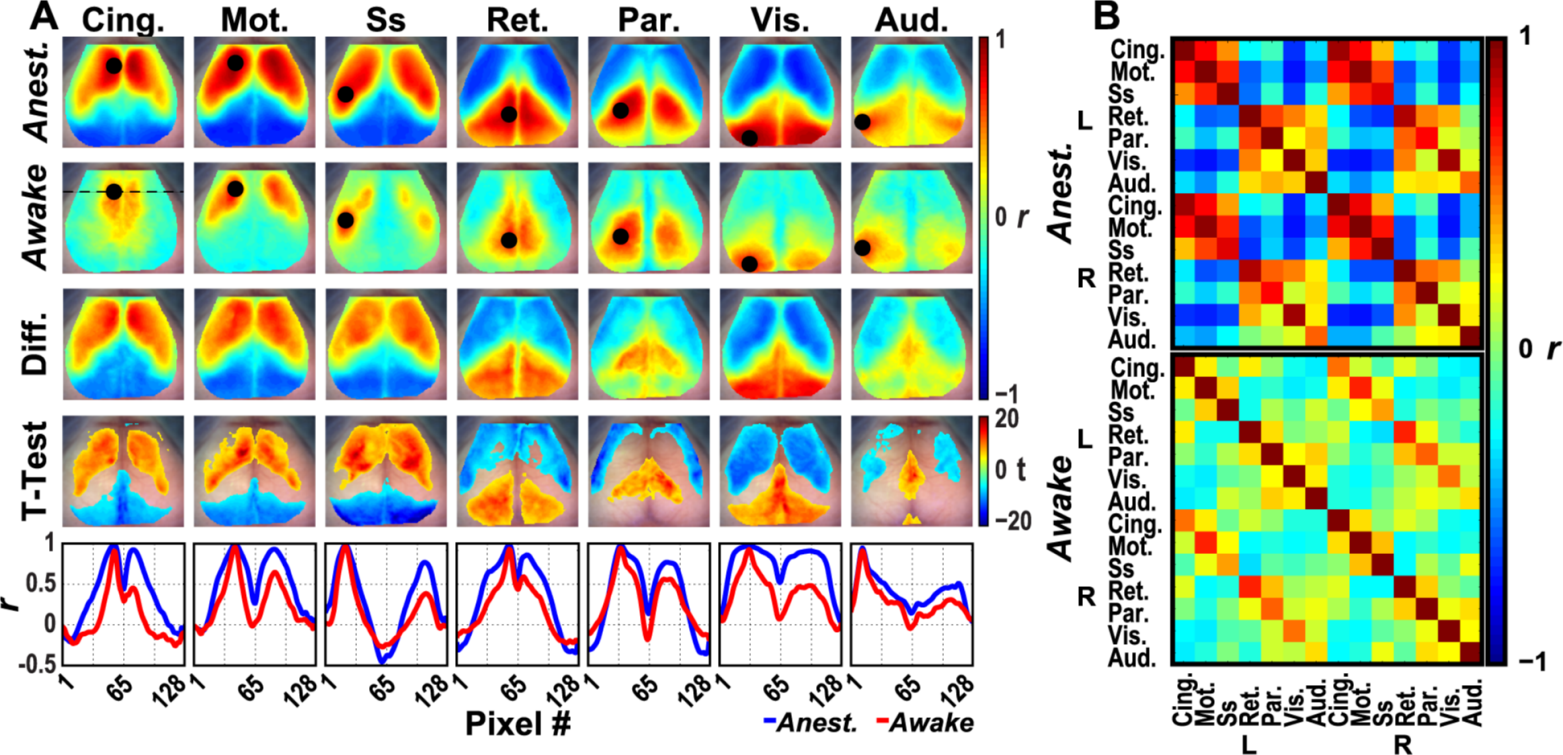
Functional connectivity mapping in GCaMP6 mice. (A) Seed-based functional connectivity maps for seven canonical functional networks in anesthetized and awake GCaMP6 mice (N = 7) at delta-band frequencies. Under anesthesia (top), delta activity (0.4–4.0Hz) drives a strongly correlated/anticorrelated network structure between anterior and posterior brain regions that is not observed in awake animals (second row). This high magnitude correlation feature is preserved in the difference images (third row) and after performing pixelwise t-tests and thresholding the t-statistic maps at a Bonferroni adjusted α = 3.1e-6 (fourth row). Together, these results show that the topography of functional connectivity maps in the anesthetized state is less sensitive to seed-based network-specific features compared to functional connectivity topography during wakefulness. Finally, horizontal line profiles through the center of each seed show higher ipsilateral and contralateral focality in the awake (red) functional connectivity maps compared to anesthetized (blue) maps (fifth row and table in S1 Table). (B) Seed-based pairwise functional connectivity matrices. Regional correlation coefficients between each seed reveal heterogeneous connectivity structure within each contrast across different states. Clusters of networks are present in the anesthetized matrix (top), including between cingulate, motor, and somatosensory networks that are diminished in the awake mouse (bottom).

To examine a potential dependence of GCaMP6 activity and functional connectivity structure on state (anesthesia vs. awake), we evaluated seed based functional connectivity maps of GCaMP6 in the higher 0.4–4.0Hz frequency band (Fig 6) given the established sleep- and anesthesia-associated phenomena that occur within it [20, 21, 39]. Under ketamine/xylazine anesthesia (Fig 6A, top row), GCaMP6 functional connectivity shows an anterior-posterior separation, with large regions of high magnitude correlations and anti-correlations across the cortex. In contrast, functional connectivity correlation structure in the awake state (Fig 6A, second row) exhibits higher spatial specificity for each network of interest. To quantify the degree of detail and localization, we thresholded using pixels with positive correlations >0.2 of the maximum correlation value in each image and then summed the remaining pixels to generate an estimate of area (here referred to as correlation area) and averaged these across all seven seeds. The mean correlation area in the awake state was 39.2% (std +/- 15.9%) smaller than that of the corresponding anesthesia maps. The difference images constructed between the group level anesthetized and awake functional connectivity maps (Fig 6A, third row) show approximately the same overall topography in cingulate, motor, somatosensory, retrosplenial, and visual functional connections. This result suggests that the awake maps have finer detail than the anesthesia maps. Further support for these state-dependent differences is seen in the pixelwise t-statistic maps (Welch’s t-test) which again are similar to the top row of Fig 6A and reinforces the structure observed in the difference images (Fig 6A, third row). Finally, horizontal line profiles through the center of each seed for both the awake (red) and anesthetized (blue) functional connectivity maps show higher focality (measured using reductions in ipsilateral and contralateral full width at half maximum, table in S1 Table.) in cingulate, motor, somatosensory, retrosplenial, and visual networks in the awake state. Visualized in matrix form (Fig 6B), the pairwise functional network connections under anesthesia (Fig 6B, top) show clustering of positive correlations in anterior networks (cingulate, motor, and somatosensory) and negative correlations in posterior networks (retrosplenial, parietal, visual, and auditory), whereas awake animals (Fig 6B, bottom) exhibit stereotypical high correlation strength only in the off-diagonals. Note the functional connectivity structure over the delta band during wakefulness is consistent across all mice (S2 Fig).

### Strong cortical delta activity is associated with anterior-posterior spontaneous GCaMP6 dynamics

**Fig 7:**
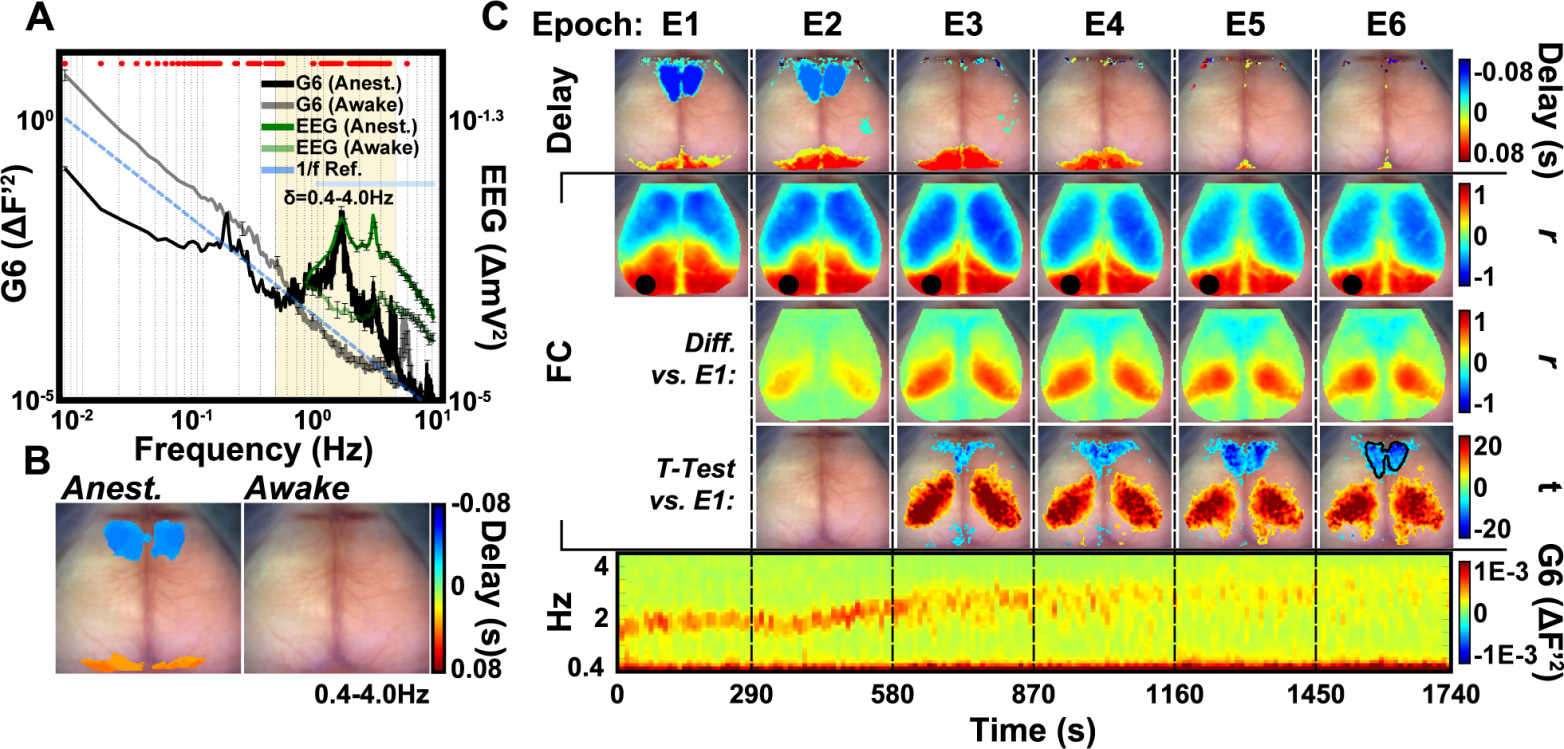
Spontaneous activity in anesthetized and awake mice reveals regional temporal delay structure. (A) Power spectra from the whole brain from a representative mouse. The presence of a peak at ~1.5Hz in both the anesthetized GCaMP6 (black) and EEG (dark green) corresponds to increased delta band (0.4–4.0Hz) activity observed in the brain under anesthesia and deep, slow-wave sleep (see Introduction). Note that this peak is absent when the mouse is awake (GCaMP6, transparent black, EEG, transparent green). Maximum cross-correlation between the whole brain signal and each pixel’s timeseries were calculated across the FOV. Cortex-wide delay topography may underlie the striking correlated/anti-correlated structure observed in the anesthetized functional connectivity maps due to the effect of phase shifts on the magnitude and sign of zero-lag correlations. Asterisks represent significant (p<0.0001) comparisons across state following two-sample Welch’s t-tests. Error bars are standard error of the mean. (B) Group-wise delay maps in anesthetized (top) and awake (bottom) mice (N = 7). The map from anesthetized mice show a temporal separation along the anterior-posterior axis of the brain, with anterior somatomotor regions leading the whole brain signal by < = 0.06s and posterior visuoparietal areas lagging the whole brain signal by < = 0.06s. This time shift is in agreement with the striking correlated/anti-correlated structure in the delta band functional connectivity maps (see Fig 4). This structure is not present in awake mice. Group-average maps are masked using pixels identified to be significant (p<0.001) following one-sample t-testing. (C) Top: Delay maps from consecutive 300s epochs from one mouse as it transitions out of anesthesia to awake reveals a loss of anterior-posterior delay structure across time. Middle section: functional connectivity maps for a left visual seed from each epoch (top row), difference images between each successive epoch and E1 (middle row), and pixelwise t-statistic maps after performing paired t-tests between E1 and each other epoch (bottom row). The boundary of the area of maximum delay in the anterior, cingulate network in the delay map in E1 (top section of C) is overlaid on the E6 t-statistic map for reference. These data show that the correlation magnitude of the parietal region differs across state and that there is agreement across delay and functional connectivity analyses in the implicated cingulate network. All analysis was performed after dividing each 300s epoch into ten 30s windows. Bottom: Spectrogram over the delta frequency band (0.4–4.0Hz) from each epoch showing the loss of delta-band activity (peak at ~1.5Hz) with time across epochs.

Compared to the functional connectivity structure of GCaMP6 during wakefulness, functional connectivity maps under anesthesia largely contain strong anti-correlations between anterior and posterior brain structures (Fig 6A). These anti-correlations might be indicative of coherent propagating waves of spontaneous GCaMP6 activity over the anterior-posterior axis. To determine if our model and system are sensitive to these dynamics, we examined the frequency content of global spontaneous GCaMP6 activity in a representative mouse under ketamine/xylazine anesthesia and while awake (Fig 7A). A phenomena commonly reported in the sleeping and anesthetized brain is the presence of prominent propagating slow-wave delta activity [40]. The GCaMP6 spectra of mice under anesthesia (Fig 7A, black trace) reveal a peak centered at ~1.5Hz that is absent in wakefulness (Fig 7A, gray trace), which is consistent with previous electrophysiological studies reporting delta-band activity [21]. Further, the state-dependence of delta activity exhibited in GCaMP6 dynamics is recapitulated in the simultaneously-acquired global field potential EEG data (anesthetized: dark green, awake: light green).

In order to better understand the observed state-dependent GCaMP6 functional connectivity structure, we examined the delay topography of the GCaMP6 signal by calculating the pixelwise lag (via cross-correlation) relative to the whole-brain signal (Fig 7B and 7C). At the group level (Fig 7B), the statewise contrast observed in Fig 6 is prominent, with somatomotor regions tending to lead the brain by up to 0.08s and visuoparietal areas lagging by a comparable interval. This temporal separation across the cortex could underlie the observed anterior/posterior structure found in the functional connectivity maps of GCaMP6 activity in anesthetized animals (Fig 6).

Finally, we evaluated spontaneous GCaMP6 activity in a mouse while it transitioned from anesthesia to wakefulness across six consecutive 5min epochs (Fig 7C). In the first epoch (E1), the mouse shows an anterior-posterior delay structure (Fig 7C, top), with cingulate regions leading the whole brain signal by ~0.08s and visual/parietal regions lagging by ~0.08s. As the mouse wakens (determined by the attenuation of delta-band power, peak at ~1.5Hz, Fig 7C spectrogram), this topography deteriorates (Fig 7C, top, transition from E2 to E3). The corresponding functional connectivity map constructed using a left visual seed is shown for each epoch (Fig 7C, second row). To improve SNR when creating average functional connectivity maps (Fig 7C, second row) and enable statistical testing using paired t-tests (Fig 7C, fourth row), each 5min epoch was divided into ten 30s windows. This window length was selected based on the observed convergence in spatial similarity of functional connectivity maps constructed using at least 30s of spontaneous data compared to the group average reference (Fig 4A). During wakefulness (Fig 7C, columns E5 & E6), the correlation maps show smaller, more detailed regions of reduced correlation magnitude, both ipsilateral and contralateral to the seed, as compared to the maps during anesthesia. Successive difference maps calculated between E1 and each subsequent epoch (E2-E6) show that connectivity in the parietal cortex preferentially changes during the transition from anesthesia (E1) to wakefulness (E6). There is also a subtle but visible difference in cingulate cortex between E1 and the later epochs. t-statistic maps (thresholded at q<0.001 after false discovery rate adjustment), constructed from pixelwise paired t-tests between E1 and all subsequent epochs, recapitulate these findings (Fig 7C, fourth row). The anterior, cingulate region leads the brain during anesthesia (E1, Fig 7C, top), which is consistent within the difference t-statistic maps of functional connectivity during the awake epochs (E5 and E6). The boundary of maximum delay value from this cingulate network (E1, Fig 7C, top) is overlaid on the t-statistic map in column E6, showing the agreement across both analysis strategies.

## Discussion

The goal of this study was to use concurrent oximetric and fluorescent GCaMP6 imaging to characterize the state-dependent differences in functional connectivity and delay structure in spontaneous calcium transients from infraslow to delta-band frequencies. For functional mapping, calcium dynamics have the appeal of being faster and more directly related to neuronal activity than hemoglobin dynamics. Specifically, we imaged spontaneous calcium activity in mice expressing GCaMP6 driven by the *Thy1* promoter. The neural origins of the fluorescence signal were established first through electrical stimulation of the hindpaw, in which the fast positive response of the corrected GCaMP6 signal to each pulse presentation indicated both the neuronal origin and also the utility of the correction for absorption transients (Fig 2). Skull-intact, chronic optical window implants area an ideal preparation because they are significantly less invasive than skull-thinning and craniotomies, reducing likelihood of confounding inflammation and edema and aiding in the minimization of animal mortality rates across time and repeat imaging sessions [41–45]. Also, there are a number of previously reported *in vivo* widefield optical neuroimaging studies in skull-intact mice [2, 15, 16] that have been successfully implemented. In a similar study, Vanni and colleagues reported a ~1.5% change in GCaMP3 fluorescence response to hindlimb stimulation, whereas in the current study we observed a GCaMP6 fluorescence increase of ~0.6% in response to hindpaw stimulation. However, it should be noted that differences in stimulation paradigm (100Hz presentations for 1s duration mechanical stimulation compared to 2Hz presentations for 4s electrical stimulation here) and imaging preparation could contribute to this observed difference. Resting GCaMP6 activity exhibited spatio-temporal correlative structure reflecting functional network connectivity within the infraslow (0.009–0.08Hz, Fig 3), intermediate (0.08–0.04Hz, Figs 3 and 5), and delta (0.4–4.0Hz, Fig 6) frequency bands. Spontaneous GCaMP6 data at this higher frequency band can provide stable, consistent functional connectivity maps with as little as 30s of imaging (Fig 4). Additionally, under resting state conditions in anesthetized mice, simultaneously-acquired EEG independently confirmed that the GCaMP6 signal contained a high-amplitude peak in delta-band that was absent in awake mice (Fig 7). Moreover, state-dependent differences in apparent functional connectivity structure in delta-band GCaMP6 activity appeared to be driven by the underlying temporal delay topography of the brain (Fig 7).

In attempting to establish a floor for the amount of spontaneous delta-band GCaMP6 data necessary to construct typical functional connectivity maps, we observed that seed-based functional connectivity maps converge on a spatially similar structure to that of group-averaged data (consisting of maps created using 300 second sessions) with as little as 30 seconds of data. We also observed, that while these 30-second windows were relatively stable (Fig 4D), there was a slight drift in the spatial similarity over five minutes. This could be due to neurophysiological changes arising across the duration of the run. However, it should be noted that this analysis does not separately examine or isolate the effects of sampling variability and physiological stability. Laumann et al. have reported that for short time windows (e.g. <300 seconds), the variance in functional connectivity Pearson correlation measurements is attributable to sampling error and is inversely proportional to time window length [46]. More recently, it has been reported that fluctuating arousal state and sleep can drive non-stationarity in functional connectivity structure, even after accounting for the variance in functional connectivity maps contributed to by motion and sampling variability [47]. The variability in the similarity of the sliding 30 second window of spontaneous GCaMP6 data (sampled from left motor cortex; Fig 4D) compared to the group average image could be driven by these artifacts. Future studies are needed to thoroughly examine the stability and stationarity of GCaMP6.

The correspondence between functional connectivity maps of HbO_2_ and GCaMP6 over the infraslow and intermediate frequency bands (Fig 5D, top and middle, S6 Fig) is in agreement with previously reported observations that measures of spontaneous calcium, local field potentials, and low frequency hemodynamics are correlated across the rat cortical vascular tree, macaque visual cortex, and the entire dorsal surface of the mouse cortex [7, 9, 33]. Collectively, these findings further reinforce the utility of functional hemodynamic measures as surrogates for neuronal activity at frequencies <0.4Hz. Interestingly, the similar functional connectivity features in the intermediate functional connectivity maps are supported by the capacity of delay-shifted GCaMP6 data to recover functional connectivity structure in spontaneous HbO_2_ data (Fig 5D, second row). Both results point to an inherent shared network topography across both calcium and hemoglobin dynamics. The intercontrast results also strongly suggest that the spontaneous calcium and hemoglobin are synchronized. An important caveat to note is the extent of overlapping mechanisms of neurovascular coupling in the evoked- and resting-states is an active area of research. It is well known that hemodynamic responses do not have a completely linear relationship with stimuli; evoked responses durations and amplitudes are nonlinearly dependent on frequency, duration, and amplitude of stimulus in addition to region-specific differences in hemodynamic response functions. Also, the differences in regulation of functional hyperemia due to spontaneous neuronal activity compared to stimulus-evoked are not known [34]. In this study, we aimed to establish the feasibility of implementing delay-shifted intercontrast functional connectivity strategies, so both seed-based, resting state delays (Fig 5, S6 Fig) as well as delays calculated using the evoked data (S7 Fig) were used. More extensive exploration of the mechanisms underlying neurovascular coupling in both resting-state and evoked conditions should be pursued in future studies.

Furthermore, the general qualitative similarity of GCaMP6 functional connectivity structure over frequency bands and across states-of-consciousness (Fig 6) suggests that there is a basis functional connectivity structure, and that at least some components of the underlying functional network architecture exist independent of state and across a wide band of frequencies. These reports add to a growing literature on stable, stereotypical functional connectivity network structure, observed using a fluorescent glutamate sensor in both isoflurane-anesthetized and awake mice at frequencies up to 12Hz [48] and using 128-channel electrocorticography in ketamine/medetomidine-anesthetized and awake macaques [49]. However, upon calculating difference images and using pixelwise statistical tests, the state-dependent changes in functional connectivity structure become clearly apparent. At the group level, seed-based functional connectivity maps created using anesthetized spontaneous data show a topography that is, to some degree, independent of network seed (Fig 6A, third and fourth row) with the clear exception of a sign change as the seeds move along the anterior-posterior axis. Across the anesthetized data, there exists a highly clustered binary structure with high magnitude correlations and anticorrelations that persists even after subtraction of the more focal and spatially specific awake maps. When examining an individual mouse transitioning from anesthesia to wakefulness, the cingulate and parietal areas change the most (Fig 7C) when using a seed placed in left visual cortex. This parietal region, located in the transition zone between the anterior and posterior network clusters, contains numerous projections to visual cortex as part of the dorsal processing stream [50] and has been shown to act as a sink for activity induced multi-modal sensory stimulation, imaged *in vivo* in mice using a voltage-sensitive dye [51]. It is possible that as a sensory integration center, it his highly synchronized with the activity of the visual cortex during anesthesia.

In addition to patterns of functional connectivity, the temporal delay topography of the cortex (Fig 7) was also dependent on state. Under anesthesia, the GCaMP6 activity over the delta frequency band propagated from anterior regions (e.g., motor) to posterior regions (e.g., visual), and is consistent with previous observations in sleeping humans and anesthetized mice [40, 52]. Using a 256-channel high-density EEG system in asleep humans (n = 8), Massimini and colleagues show that each cycle of this slow oscillation is a traveling wave that originates in prefrontal-orbitofrontal regions and propagates along the anterior-posterior axis, sweeping the cortex [40]. At the group level (Fig 7B, left), the total time needed for this wave to traverse the cortex was ~160ms which is at parity with the time necessary for delta waves originating in orbitofrontal regions to propagate posteriorly in humans (~120-200ms) [40].

These travelling oscillatory delta waves have been suggested to facilitate memory consolidation in the context of slow wave, natural sleep [53]. This neuronal oscillation consists of alternation between periods of membrane potential hyperpolarization (“down states”, a period of neuronal silence) and depolarizing envelopes (“up states”), first described by Steriade and colleagues in cats using intracellular recordings [21]. In natural sleep, these oscillations occur at approximately 0.4Hz, but can be pharmacologically modulated. For example, in the presence of ketamine/xylazine, the duration of the depolarizing “up” envelope was reduced, increasing the overall frequency of oscillation to ~1Hz. Importantly, ketamine and xylazine, used in the present study, has been shown to best mimic natural sleep, especially in the context of the sleep slow oscillation, compared to other common anesthetics [20]. The strength of the oscillations likely underlies the strong correlations and anti-correlations seen in the functional connectivity maps of the delta frequency band (Fig 6A). The temporal separation observed across the cortex drives activity in anterior somatomotor networks to be more out of phase with posterior visual/parietal regions (Fig 7C), increasing the apparent strength in contralateral homotopic connections while also increasing the anti-correlation magnitudes between somatosensory and parietal regions. Human fcMRI studies have shown that functional connectivity structure can be a consequence of the delay topography while the reverse might not necessarily hold true [54]. It is therefore possible that this prominent propagating wave of delta-band oscillations is superimposed on spontaneous activity associated with functional network connectivity structure (e.g. that seen in wakefulness), and provides the increased correlation magnitudes and anterior-posterior clustering unobserved in awake animals. This concept is supported by recent work in isoflurane-anesthetized transgenic *Emx1*/GCaMP3 mice in which traveling transient neuronal coactivations occurred in areas exhibiting high functional connectivity [55]. In this study, we observed that the same anterior, cingulate regions that lead the brain in the delay maps (Fig 7C, top, E1 & E2) are also the regions of maximal difference in the functional connectivity structure across a transition from anesthesia to wakefulness (Fig 7C, middle section: bottom, E5 & E6). Interestingly, the delay structure present over the delta band under anesthesia was not observed in awake animals (Fig 7). However, the current analysis was limited by the sampling rate (16.8Hz) and would not be sensitive to delays shorter than approximately 0.06 seconds.

While there are studies of evoked responses with macroscopic imaging of GECIs, there are relatively few with GECI’s that explore either functional connectivity [8, 15, 16, 55] or other analysis of calcium dynamics(e.g. functional hyperemia) [56]. Of the studies reporting correlative functional structure in spontaneous calcium dynamics, they are either limited to one state of consciousness and/or to poorer performing GECIs [8, 15, 55]. Collectively, though, these previous reports largely support the results of the present study. For example, Vanni et al. demonstrated mesoscale somatomotor functional connections in spontaneous calcium activity, using an isoflurane-anesthetized *Emx1*/GCaMP3 mouse model, which resemble the somatosensory and motor seed-based functional connectivity maps of the present study [15]. Recently, Silasi and colleagues reported similar homotopic functional connectivity structure in seed-based maps in awake and isoflurane-anesthetized mice. They observed an increase in network focality and reduction in correlation magnitude upon wakefulness, analogous to the results reported here, despite the difference in selected anesthesia (isoflurane vs. ketamine/xylazine), frequency content of spontaneous data (1-10Hz vs. 0.4–4.0Hz), and amount of data included in analysis (approximately 35min of awake imaging data from 7 mice and approximately 10min of anesthetized data from 2 mice compared to the present study with 130 minutes of anesthetized data and 225min of awake data across 7 mice, acquired within the same imaging session) [16].

There are several limitations to the current study that indicate the need for further work. While the GCaMP6 fluorescence emission ratiometric correction method implemented here removes the negative trend in the uncorrected raw fluorescence signal (left hindpaw stimulation, Fig 2C, top). The slow decay of the corrected signal possibly indicates overcorrection, though this may be apparent due to the strong stimulation. Other methods, such as including a diffuse reflectance measurement of the excitation wavelength, estimating the differential absorption coefficient of the excitation wavelength using the calculated changes in hemoglobin concentrations, or empirically deriving system-specific optimizations in principal can provide more accurate corrections [57, 58] and likely would reduce the slow decay. Thus, these correction methods remain a useful and active area of research. In addition, S4 Fig contains spontaneous GCaMP6 time courses before and after ratiometric correction along with HbO_2_ and HbR time traces. Using spontaneous data to compare correction metrics proves more difficult than hindpaw stimulation because of the lack of features present to evaluate correction efficacy. However, S4 Fig, panel C shows anesthesia-associated slow wave oscillations and there is little evidence for HbO_2_ or HbR contamination following ratiometric correction; he GCaMP6 corrected (black) and uncorrected (teal) traces behave similarly. For example, to remove confounding absorption of *in situ* fluorescence by glycation end-products in porcine skin models of diabetes, Hull and colleagues estimated two scaling parameters used in their ratiometric correction that minimized intra-subject fluorescence variability across sessions and maximized the expected correlation between age and fluorescence intensity [57]. In addition to the slow wave peak at ~1.5Hz in the anesthetized state, there are also small fairly narrow peaks observed in the awake and anesthetized spontaneous GCaMP6 frequency spectra at approximately 3-5Hz and 8-10Hz. While observable on a log scale after significant data averaging, both peaks have four orders of magnitude lower power compared to the low frequency fluctuations at 0.01Hz and more than one order of magnitude lower power compared to the slow wave peak at ~1.5Hz. Given that the GCaMP6f sensor used in this study has a frequency cut-off around 2-4Hz [26], it is likely that the peaks observed in the awake and anesthetized spontaneous GCaMP6 frequency spectra (Fig 7A) at approximately 4Hz and 8Hz are not due to GCaMP6 dynamics. Rather they might be either small aliasing artifacts above the camera frame rate, or physiological in nature since mouse respiration is ~3-5Hz and heart rate is ~8-10Hz [2]that are not completely removed from the corrected GCaMP6 images. However, due to the variance and width of the anesthesia-dependent delta-band peak across and the variance of the frequency of respiration across mice, using a decade-wide frequency band (0.4–4.0Hz) consistently for all mice sufficiently captured the state-dependent frequency content driving the results of this study. Additionally, flavoproteins are auto fluorescing metabolites that display elevated fluorescence emission with increasing oxidative metabolism [59]. Problematically, their fluorescence spectra overlap with that of GCaMP6 (excitation at λ = 460nm and emission at λ = 520nm). However, their temporal dynamics are distinctly slower than those of calcium. In particular, it has been shown that compared to flavoprotein auto fluorescence, GCaMP3 has a significantly faster onset (GCaMP3: 0.28s vs. flavoproteins: 0.62s; *p* = 0.002) and time to peak (GCaMP3: 0.65s vs flavoprotein: 1.11s; *p*<0.001) in response to a 1s hindlimb stimulation at 100Hz [15]. Similarly, *in vitro* studies in rat cortical slices showed an evoked calcium response (measured using the rhod-2 calcium indicator) to 1s 20Hz electrical stimulation peaking ~1.5s before the measured flavoprotein auto fluorescent response [60]. Thus, the slower temporal characteristics of flavoprotein dynamics compared to calcium transients, at least over higher frequencies (e.g. delta-band analyses in this study), will likely result in little contamination of the GCaMP6 signal. Furthermore, given the emphasis of this study on the higher-frequency delta band in examining the effects of ketamine/xylazine anesthesia compared to wakefulness on functional connectivity, slower hemodynamic oscillations will have less correlative structure at these frequencies, as demonstrated by recent work that showed peak correlation between spontaneous GCaMP6f and HbT in awake mice at 0.21Hz that had reduced ~30% by ~0.8Hz (32). Finally, *Thy1* expression patterns determine the effective field-of-view of our imaging system and can vary with age, sex, filial generation, and genetic background [61]. The cohort of mice in the present study share the same background and are matched for age and sex. However, mice were either F1 (n = 3) or F2 (n = 4) relative to their F0 breeding pair from The Jackson Laboratory (https://www.jax.org/). Despite this difference, study results were assessed at the individual mouse level and were qualitatively recapitulated across the group.

Future studies could take advantage of concurrent GCaMP6 and hemodynamic imaging to study diseases that impact neurovascular coupling by providing evidence on the sequence of neurodegeneration and vasculopathy underlying these conditions. In this study, we found a high concordance between calcium and HbO_2_ at infraslow and intermediate frequencies. These findings were in healthy adult mice in which neurovascular coupling is relatively intact and spatially homogenous across the cortex. However, in a wide variety of diseases such as stroke [3, 62] and Alzheimer’s disease (AD) [4, 63, 64], neurovascular coupling and hemodynamic functional connectivity can be compromised or modulated. For example, in a mouse model of AD, a decline in hemodynamic functional connectivity was found to be driven, in part, by deposition of amyloid-β (Aβ) plaques[4]. This AD-associated functional disruption was restored upon immunotherapy with HJ6.3 antibody that limits formation and growth of new Aβ plaques [5], with treated mice showing mildly improved performance in a spatial learning task. A concurrently-acquired imaging probe of neuronal activity (e.g. GCaMP6) could provide mechanistic information underlying these observed cognitive changes. Furthermore, AD-related Aβ accumulation and deposition has been recently shown to disrupt delta-band slow wave coherence and propagation [65], providing a fitting target of study for the multi-modal imaging system described here.

In summary, GCaMP6 activity reveals functional connectivity structure over a frequency range (from 0.009Hz to >4Hz) higher than the canonical functional connectivity band (0.009 to 0.08Hz) that is state-dependent. Calcium-based spontaneous mapping of functional connectivity is faster and more directly coupled to neural activity than hemoglobin-based mapping of functional connectivity. Further, the calcium signal can be targeted and localized to specific types of cells providing an exquisitely specific method for detailed mapping of mesocopic connectivity patterns. Finally, combined GCaMP6 and hemodynamic imaging will enable the dissociation of changes in ionic- and hemodynamic-based networks and neurovascular coupling and provide a framework for subsequent studies of neurological disease such as stroke.

## Supporting information

S1 Fig Hemodynamic functional connectivity maps and matrices. Group-level functional connectivity maps (A) and matrices (B) of infraslow (0.009–0.08Hz) spontaneous oxyhemoglobin (HbO_2_), deoxyhemoglobin (HbR), and total hemoglobin (HbT) acquired in the ketamine/xylazine-anesthetized state. The same canonical network seeds used in Figs 4 and 5 were applied here.
(TIF)

S2 Fig GCaMP6 functional connectivity maps from individual mice. GCaMP6 functional connectivity maps of all 7 individual mice in the imaging cohort, filtered over the delta (0.4–4.0Hz) frequency band. The same canonical network seeds used in Figs 4 and 5 were applied here. Data shown are acquired in the awake state.
(TIF)

S3 Fig Mouse brain atlas of cortical functional regions. Using the Paxinos histological atlas [29], an atlas of the functional areas of the mouse cortex was constructed. Each of the seven network seeds used in all seed-based analysis in this study are color-coded to their respective region of the atlas (Red: motor; Yellow: cingulate; Green: Somatosensory; Magenta: retrosplenial; Lavender: parietal; Pink: auditory; Blue: visual). Adapted from White & Bauer et al., 2011.
(TIF)

S4 Fig Functional connectivity traces in awake and anesthetized mice. GCaMP6 raw, GCaMP corrected, HbO_2_, and HbR contrasts are all shown for the right motor cortex time trace in awake and anesthetized mice across the 0.009–0.08Hz, 0.08–0.4Hz, and 0.4–4.0Hz frequency bands.
(TIF)

S5 Fig Stability and stationarity of spontaneous GCaMP6 activity in functional connectivity infraslow analysis. Functional connectivity maps were constructed using time windows of spontaneous 0.009–0.08Hz GCaMP6 (awake) data of increasing length from one mouse (Mouse 2). Windows were increased in length by ~2.6s up to ~300s. Spatial similarity (Dice coefficient) was calculated between each of these functional connectivity maps for all networks relative to their corresponding group level map. Across cingulate and motor networks, similarity scores converge at 0.9 with a window length of ~30s. However, other networks in this frequency band remain unstable and do not converge to high dice coefficients during windows as long as 300s.
(TIF)

S6 Fig Infraslow intercontrast functional connectivity mapping using spontaneous delays. HbO_2_ and HbR data is shifted based on the correlation calculated between the spontaneous hemoglobin data and spontaneous GCaMP6 data. Seed-based group-averaged functional connectivity mapping in filtered 0.009–0.08Hz anesthetized data using HbO_2_, GCaMP6, and HbR and cross-correlation traces (with mean delays of maximum correlation in black text with standard errors of the mean in parenthesis) calculated between GCaMP6 and HbO_2_ (red lines) or GCaMP6 and HbR (blue lines).
(TIF)

S7 Fig Intercontrast functional connectivity mapping using evoked delays. HbO_2_ and HbR data is shifted based on the correlation calculated between the evoked hemoglobin data and evoked GCaMP6 data (see Fig 5B for calculated -0.65s evoked shift). Seed-based group-averaged functional connectivity mapping in filtered (A) 0.009–0.08Hz and (B) 0.08–0.4Hz anesthetized data using HbO_2_, GCaMP6, and HbR.
(TIF)

S1 Table State-dependent changes in seed-based functional connectivity network widths. (DOCX)

S2 Table Spatial similarity between either HbO_2_ or HbR and GCaMP6 seed-based functional connectivity maps from anesthetized mice. (DOCX)

S3 Table Spatial similarity between either HbO_2_ or HbR delay-shifted (spontaneous and evoked) intercontrast seed-based functional connectivity maps and GCaMP6 seed-based functional connectivity maps from anesthetized mice. (DOCX)
