# Supplementary figures and images for: Functional connectivity structure of cortical calcium dynamics in anesthetized and awake mice
Source: PLoS One. 2017 Oct 19;12(10):e0185759. doi: 10.1371/journal.pone.0185759 (PMC5648115; doi:10.1371/journal.pone.0185759)

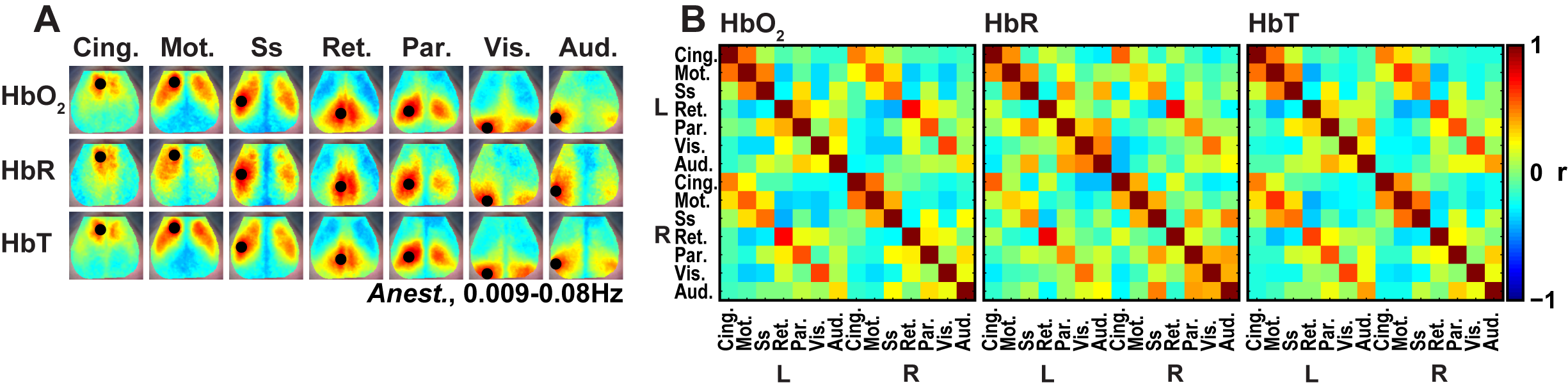

Supplement: S1 Fig — Group-level functional connectivity maps (A) and matrices (B) of infraslow (0.009–0.08Hz) spontaneous oxyhemoglobin (HbO2), deoxyhemoglobin (HbR), and total hemoglobin (HbT) acquired in the ketamine/xylazine-anesthetized state. The same canonical network seeds used in Figs 4 and 5 were applied here. (TIF) [file pone.0185759.s001.tif]

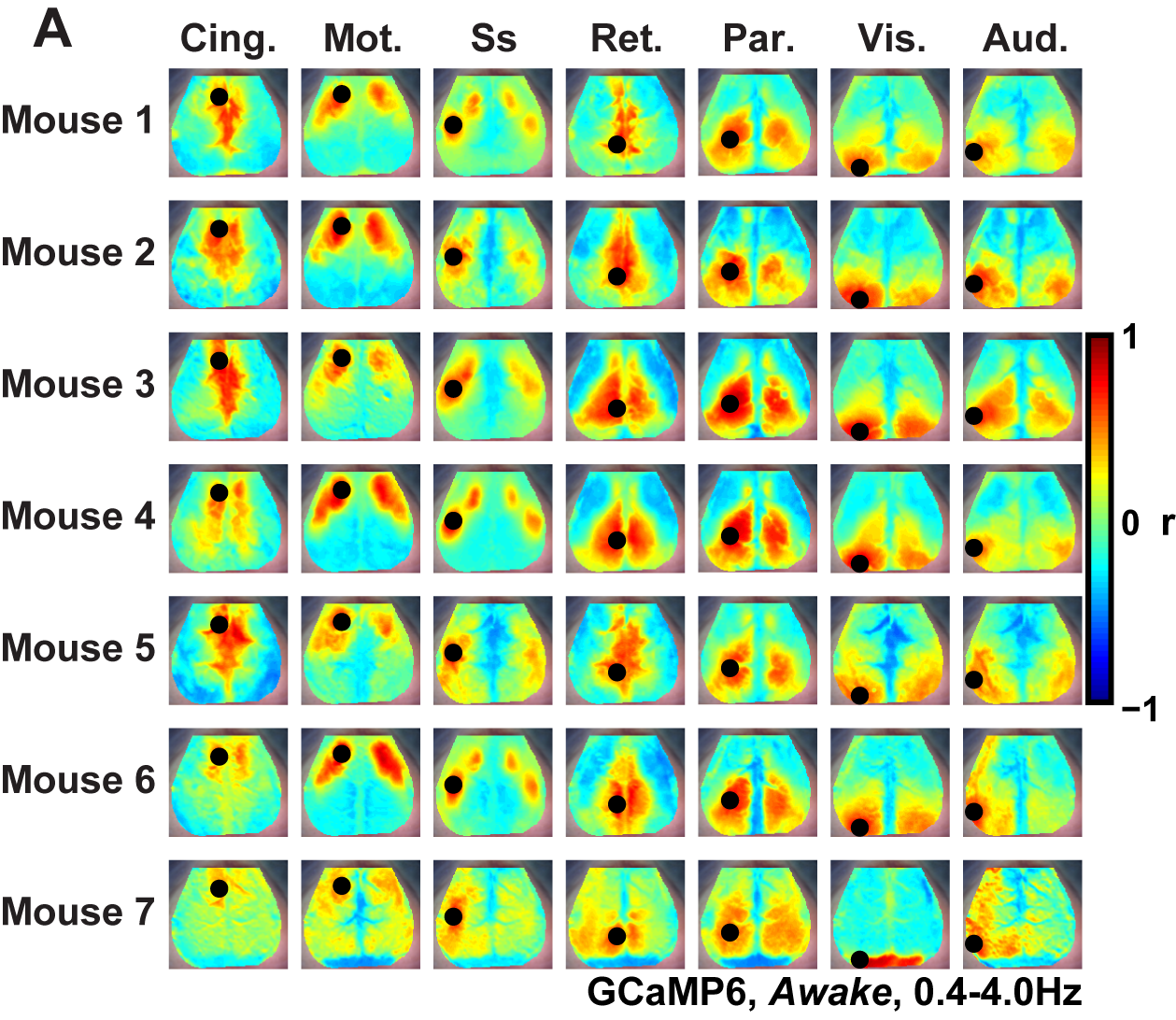

Supplement: S2 Fig — GCaMP6 functional connectivity maps of all 7 individual mice in the imaging cohort, filtered over the delta (0.4–4.0Hz) frequency band. The same canonical network seeds used in Figs 4 and 5 were applied here. Data shown are acquired in the awake state. (TIF) [file pone.0185759.s002.tif]

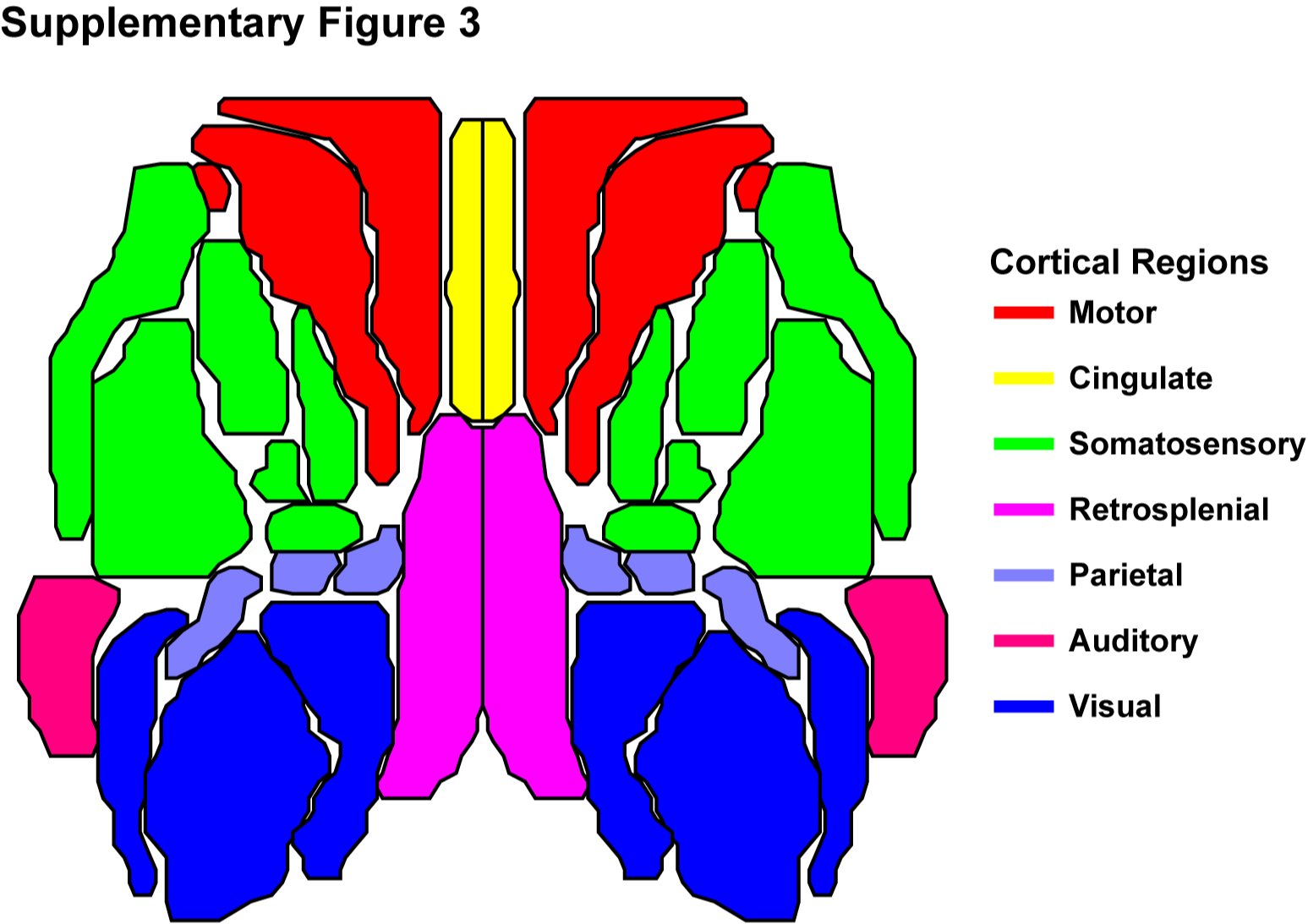

Supplement: S3 Fig — Using the Paxinos histological atlas [29], an atlas of the functional areas of the mouse cortex was constructed. Each of the seven network seeds used in all seed-based analysis in this study are color-coded to their respective region of the atlas (Red: motor; Yellow: cingulate; Green: Somatosensory; Magenta: retrosplenial; Lavender: parietal; Pink: auditory; Blue: visual). Adapted from White & Bauer et al., 2011. (TIF) [file pone.0185759.s003.tif]

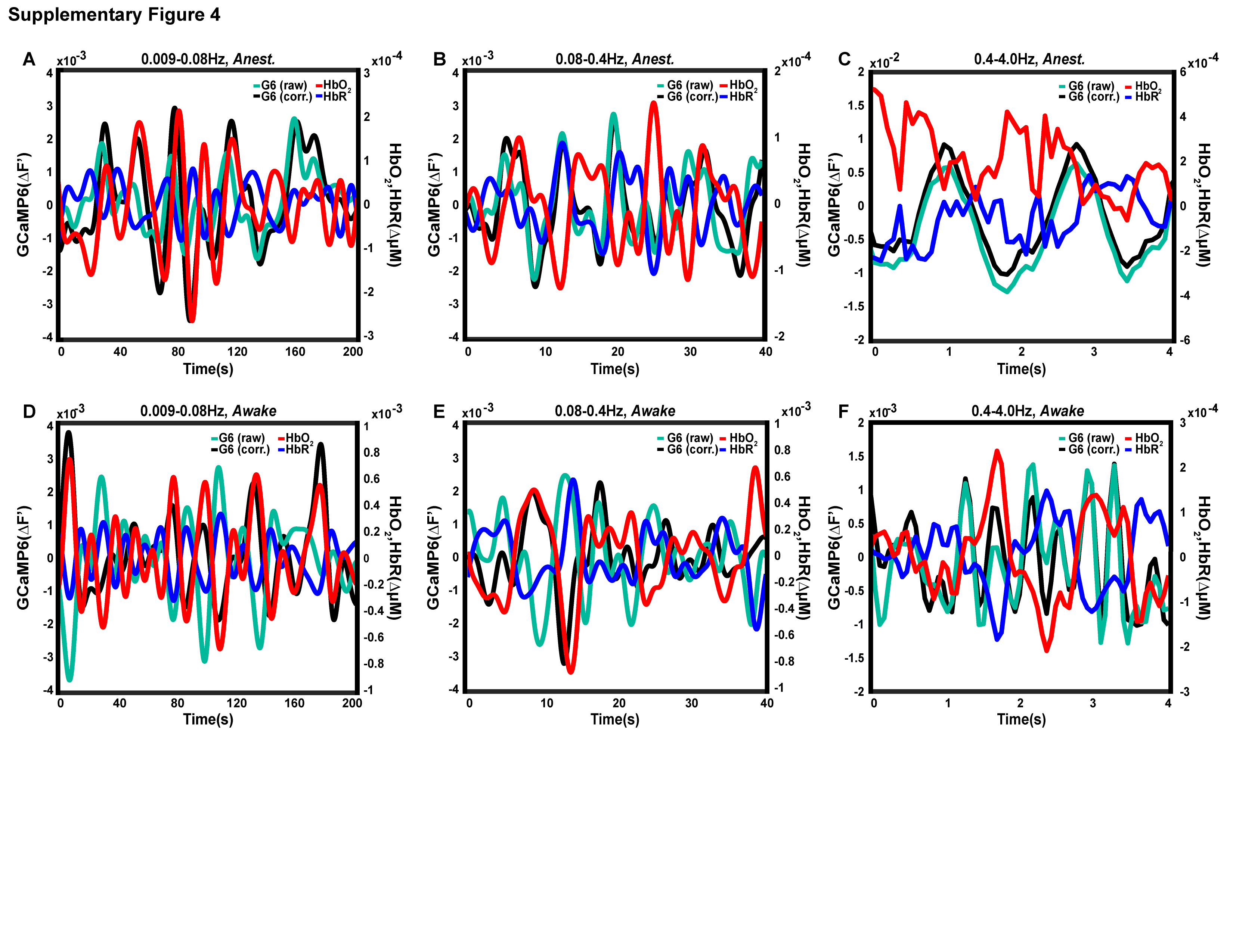

Supplement: S4 Fig — GCaMP6 raw, GCaMP corrected, HbO2, and HbR contrasts are all shown for the right motor cortex time trace in awake and anesthetized mice across the 0.009–0.08Hz, 0.08–0.4Hz, and 0.4–4.0Hz frequency bands. (TIF) [file pone.0185759.s004.tif]

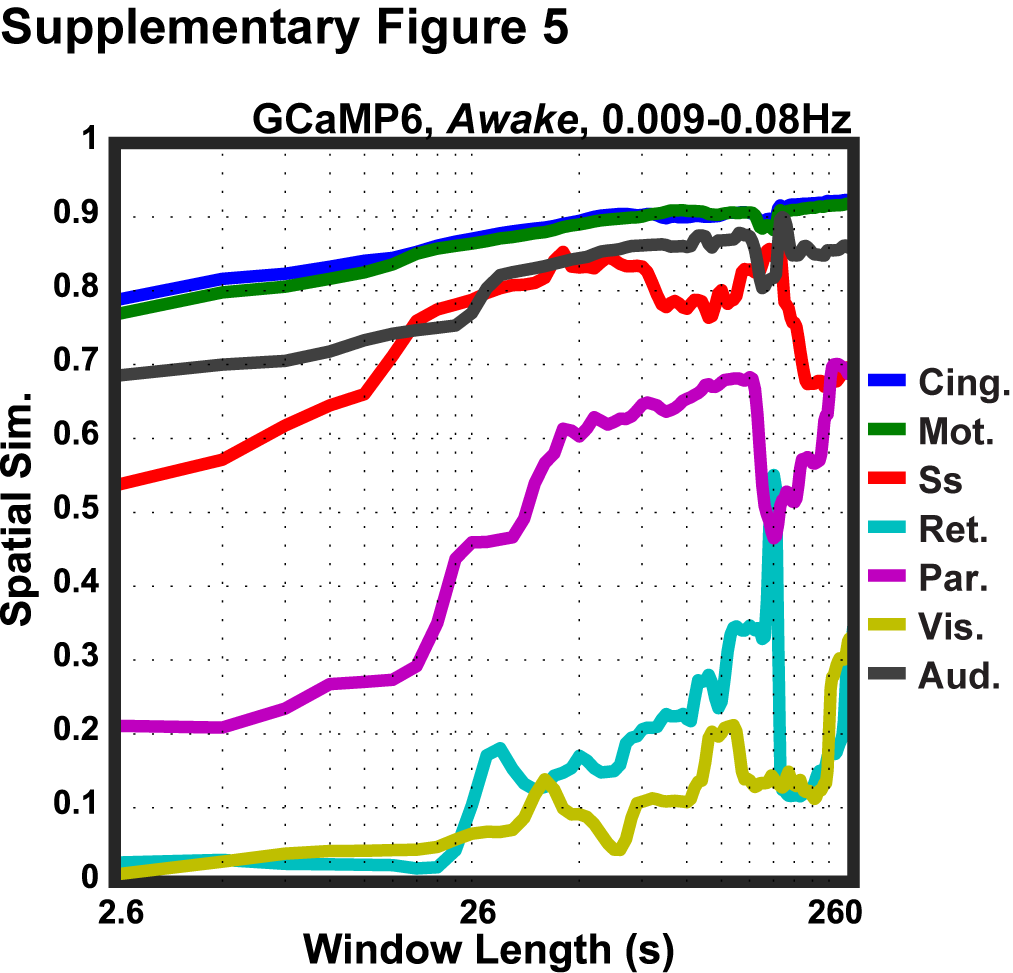

Supplement: S5 Fig — Functional connectivity maps were constructed using time windows of spontaneous 0.009–0.08Hz GCaMP6 (awake) data of increasing length from one mouse (Mouse 2). Windows were increased in length by ~2.6s up to ~300s. Spatial similarity (Dice coefficient) was calculated between each of these functional connectivity maps for all networks relative to their corresponding group level map. Across cingulate and motor networks, similarity scores converge at 0.9 with a window length of ~30s. However, other networks in this frequency band remain unstable and do not converge to high dice coefficients during windows as long as 300s. (TIF) [file pone.0185759.s005.tif]

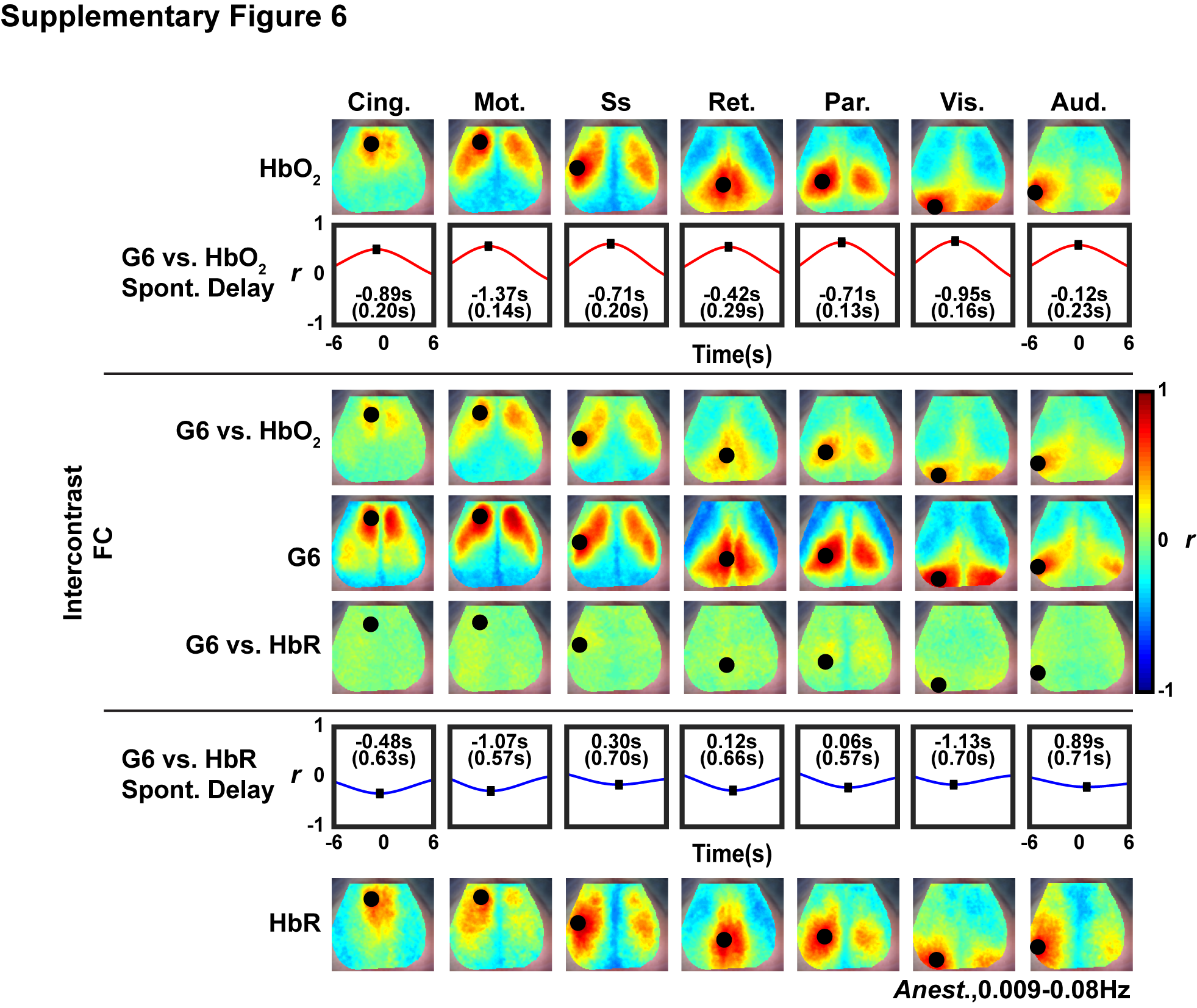

Supplement: S6 Fig — HbO2 and HbR data is shifted based on the correlation calculated between the spontaneous hemoglobin data and spontaneous GCaMP6 data. Seed-based group-averaged functional connectivity mapping in filtered 0.009–0.08Hz anesthetized data using HbO2, GCaMP6, and HbR and cross-correlation traces (with mean delays of maximum correlation in black text with standard errors of the mean in parenthesis) calculated between GCaMP6 and HbO2 (red lines) or GCaMP6 and HbR (blue lines). (TIF) [file pone.0185759.s006.tif]

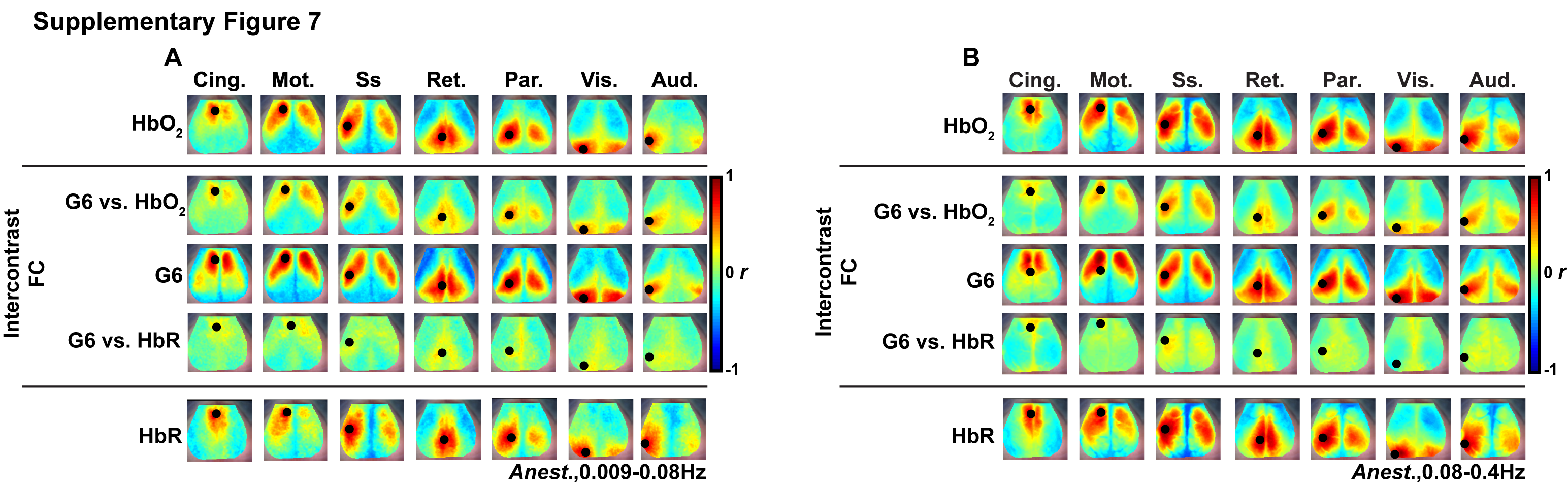

Supplement: S7 Fig — HbO2 and HbR data is shifted based on the correlation calculated between the evoked hemoglobin data and evoked GCaMP6 data (see Fig 5B for calculated -0.65s evoked shift). Seed-based group-averaged functional connectivity mapping in filtered (A) 0.009–0.08Hz and (B) 0.08–0.4Hz anesthetized data using HbO2, GCaMP6, and HbR. (TIF) [file pone.0185759.s007.tif]
