# Supplementary material for: Functional connectivity structure of cortical calcium dynamics in anesthetized and awake mice
Source: PLoS One. 2017 Oct 19;12(10):e0185759. doi: 10.1371/journal.pone.0185759 (PMC5648115; doi:10.1371/journal.pone.0185759)
Supplement: S1 Table — (DOCX) [file pone.0185759.s008.docx]

Supplemental Table 1. State-dependent changes in seed-based functional connectivity network widths.

|  | **Ipsilateral FWHM** | |  | **Contralateral FWHM** | |  |
| --- | --- | --- | --- | --- | --- | --- |
| **Network** | Anest. | Awake | P-value^a^ | Anest. | Awake | P-value^a^ |
| **Cing**. | 975 (155) | 643 (78) | 5.7e-12* | 1362 (185) | 1009 (252) | 2.3e-09* |
| **Mot**. | 1005 (157) | 726 (88) | 4.4e-10* | 1872 (258) | 1595 (328) | 0.00010* |
| **Ss** | 1116 (166) | 792 (64) | 8.5e-11* | 1743 (341) | 1518 (500) | 0.014 |
| **Ret.** | 966 (273) | 751 (180) | 0.00046* | 1860 (451) | 1421 (546) | 0.00028* |
| **Par.** | 1269 (425) | 1050 (334) | 0.015 | 2157 (432) | 2217 (601) | 0.314 |
| **Vis.** | 2727 (712) | 1572 (687) | 9.1e-09* | 3561 (509) | 3096 (410) | 0.00013* |
| **Aud.** | 900 (429) | 801 (141) | 0.131 | 2712 (1007) | 2356 (748) | 0.062 |

FWHM, full width at half maximum.

Mean (SD) in µm are shown.

^a^p-values determined using Welch’s T-test.

*Significant difference using a Bonferonni-corrected α=0.007 threshold.
