# Supplementary material for: Functional connectivity structure of cortical calcium dynamics in anesthetized and awake mice
Source: PLoS One. 2017 Oct 19;12(10):e0185759. doi: 10.1371/journal.pone.0185759 (PMC5648115; doi:10.1371/journal.pone.0185759)
Supplement: S2 Table — (DOCX) [file pone.0185759.s009.docx]

Supplemental Table 2. Spatial similarity between either HbO_2_ or HbR and GCaMP6 seed-based functional connectivity maps from anesthetized mice.

|  | **0.009-0.08Hz** | | **0.08-0.4Hz** | |
| --- | --- | --- | --- | --- |
| **Network** | HbO_2_ | HbR | HbO_2_ | HbR |
| **Cing**. | 0.45 (0.22) | 0.33 (0.16) | 0.52 (0.20) | 0.44 (0.18) |
| **Mot**. | 0.57 (0.17) | 0.41 (0.15) | 0.61 (0.19) | 0.56 (0.18) |
| **Ss** | 0.60 (0.16) | 0.42 (0.17) | 0.66 (0.13) | 0.60 (0.13) |
| **Ret.** | 0.58 (0.18) | 0.49 (0.18) | 0.65 (0.16) | 0.59 (0.18) |
| **Par.** | 0.60 (0.11) | 0.43 (0.12) | 0.62 (0.16) | 0.55 (0.17) |
| **Vis.** | 0.60 (0.15) | 0.43 (0.19) | 0.68 (0.13) | 0.60 (0.20) |
| **Aud.** | 0.41 (0.21) | 0.32 (0.21) | 0.56 (0.21) | 0.45 (0.19) |

Mean (SD) are shown.

Spont., spontaneous data
