# Supplementary material for: Functional connectivity structure of cortical calcium dynamics in anesthetized and awake mice
Source: PLoS One. 2017 Oct 19;12(10):e0185759. doi: 10.1371/journal.pone.0185759 (PMC5648115; doi:10.1371/journal.pone.0185759)
Supplement: S3 Table — (DOCX) [file pone.0185759.s010.docx]

Supplemental Table 3. Spatial similarity between either HbO_2_ or HbR delay-shifted (spontaneous and evoked) intercontrast seed-based functional connectivity maps and GCaMP6 functional connectivity maps from anesthetized mice (see Fig 5).

|  | **Spont., 0.009-0.08Hz** | | **Spont., 0.08-0.4Hz** | | **Evoked, 0.009-0.08Hz** | | **Evoked, 0.08-0.4Hz** | |
| --- | --- | --- | --- | --- | --- | --- | --- | --- |
| **Network** | HbO_2_ | HbR | HbO_2_ | HbR | HbO_2_ | HbR | HbO_2_ | HbR |
| **Cing**. | 0.45 (0.27) | 0.0079 (0.18) | 0.51 (0.22) | 0.22 (0.21) | 0.45 (0.28) | 0.21 (0.25) | 0.44 (0.15) | 0.15 (0.15) |
| **Mot**. | 0.51 (0.23) | 0.015 (0.19) | 0.64 (0.14) | 0.34 (0.16) | 0.50 (0.23) | 0.23 (0.19) | 0.49 (0.21) | 0.12 (0.28) |
| **Ss** | 0.57 (0.22) | 0.17 (0.19) | 0.67 (0.095) | 0.47 (0.12) | 0.57 (0.22) | 0.26 (0.22) | 0.59 (0.20) | 0.28 (0.32) |
| **Ret.** | 0.51 (0.27) | 0.048 (0.19) | 0.59 (0.20) | 0.30 (0.23) | 0.50 (0.27) | 0.30 (0.29) | 0.51 (0.21) | 0.24 (0.23) |
| **Par.** | 0.53 (0.24) | 0.064 (0.19) | 0.61 (0.15) | 0.31 (0.17) | 0.53 (0.28) | 0.21 (0.28) | 0.54 (0.19) | 0.23 (0.16) |
| **Vis.** | 0.51 (0.24) | 0.11 (0.24) | 0.65 (0.13) | 0.39 (0.14) | 0.50 (0.24) | 0.17 (0.30) | 0.52 (0.20) | 0.043 (0.28) |
| **Aud.** | 0.37 (0.29) | 0.11 (0.20) | 0.59 (0.20) | 0.38 (0.18) | 0.36 (0.30) | 0.081 (0.23) | 0.52 (0.23) | 0.18 (0.27) |

Mean (SD) are shown.

Spont., spontaneous data
